# Crystallographic structure of a small molecule SIRT1 activator-enzyme complex

**DOI:** 10.1038/ncomms8645

**Published:** 2015-07-02

**Authors:** Han Dai, April W. Case, Thomas V. Riera, Thomas Considine, Jessica E. Lee, Yoshitomo Hamuro, Huizhen Zhao, Yong Jiang, Sharon M. Sweitzer, Beth Pietrak, Benjamin Schwartz, Charles A. Blum, Jeremy S. Disch, Richard Caldwell, Bruce Szczepankiewicz, Christopher Oalmann, Pui Yee Ng, Brian H. White, Rebecca Casaubon, Radha Narayan, Karsten Koppetsch, Francis Bourbonais, Bo Wu, Junfeng Wang, Dongming Qian, Fan Jiang, Cheney Mao, Minghui Wang, Erding Hu, Joe C. Wu, Robert B. Perni, George P. Vlasuk, James L. Ellis

**Affiliations:** 1 Sirtris, a GlaxoSmithKline Company, 200 Technology Square, Suite 300, Cambridge, Massachusetts 02139, USA; 2 GlaxoSmithKline, 1250S. Collegeville Road, Collegeville, Pennsylvania 19426, USA; 3 ExSAR Corporation, 11 Deer Park Drive, Suite 103, Monmouth Junction, New Jersey 08852, USA; 4 High Magnetic Field Laboratory, Hefei Institutes of Physical Science, Chinese Academy of Sciences, 350 Shushanhu Road, Hefei, Anhui Province 230031, China; 5 Viva Biotech, 334 Aidisheng Road, Zhangjiang High-tech Park, Shanghai 201203, China

## Abstract

SIRT1, the founding member of the mammalian family of seven NAD^+^-dependent sirtuins, is composed of 747 amino acids forming a catalytic domain and extended N- and C-terminal regions. We report the design and characterization of an engineered human SIRT1 construct (mini-hSIRT1) containing the minimal structural elements required for lysine deacetylation and catalytic activation by small molecule sirtuin-activating compounds (STACs). Using this construct, we solved the crystal structure of a mini-hSIRT1-STAC complex, which revealed the STAC-binding site within the N-terminal domain of hSIRT1. Together with hydrogen-deuterium exchange mass spectrometry (HDX-MS) and site-directed mutagenesis using full-length hSIRT1, these data establish a specific STAC-binding site and identify key intermolecular interactions with hSIRT1. The determination of the interface governing the binding of STACs with human SIRT1 facilitates greater understanding of STAC activation of this enzyme, which holds significant promise as a therapeutic target for multiple human diseases.

## Abstract (web summary)

Sirtuins are NAD^+^-dependent deacylases implicated in the regulation of stress responses, bioenergetics and epigenetic control. Here the authors describe the crystal structure of a sirtuin-activating compounds (STAC)-sirtuin complex and begin to elucidate the mechanism of sirtuins activation by STACs.

## Graphical abstract

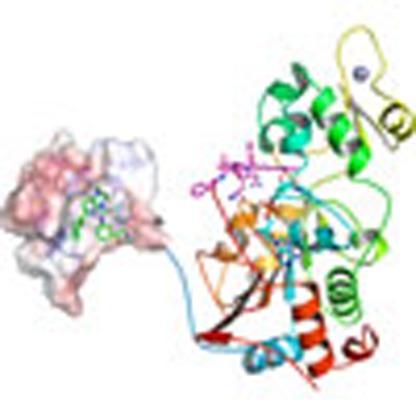

Sirtuins are a family of highly conserved NAD^+^-dependent deacylases that have been linked to a number of important biological processes across a broad span of diverse organisms such as *Saccharomyces cerevisiae*, *Caenorhabditis elegans*, *Drosophilla melanogaster* and *Mus musculus*, among others^1^,^2^. Sirtuins generally catalyze the deacylation of modified lysine residues in protein substrates coupled with the breakdown of NAD^+^ into nicotinamide (NAM) and 2'-*O*-acyl-ADP-ribose. Of the seven sirtuins (SIRT1-7) that have been identified in mammals^3^, human SIRT1 (hSIRT1) is the most studied isoform, and has been shown to be regulated by calorie restriction and to be involved in multiple biological processes^4^,^5^,^6^,^7^. The validated, protective role of increased mammalian SIRT1 activity in metabolic disorders^8^, neurodegeneration^9^ and inflammation^10^,^11^ makes this enzyme an attractive therapeutic target. To this end, the development of pharmacological approaches to increase the enzymatic activity of hSIRT1 might lead to a new generation of therapeutic agents for a wide spectrum of diseases associated with aging. Small molecule sirtuin-activating compounds (STAC) have been developed which increase the catalytic deacetylation of specific Lys residues by hSIRT1 in multiple substrates, resulting in a variety of biological responses^12^,^13^,^14^. However, the molecular mechanism of hSIRT1 activation by STACs remains controversial. Questions as to whether STACs directly activate hSIRT1 persist^15^ despite evidence of allosteric activation^13^. Recently, a single point mutation of the Glu^230^ residue of hSIRT1 has been shown to attenuate kinetic activation by STACs^16^, further demonstrating a direct effect on hSIRT1. Structural characterizations of hSIRT1 fragments have shed light on the inhibitor binding and key regulatory element^17^,^18^. Similar to other sirtuins, hSIRT1 catalytic domain contains a Rossmann-fold large lobe and a zinc-binding small lobe and undergoes a significant conformational change of domain closure upon substrate/ligand occupying the active site^19^,^20^,^21^. However, the molecular details governing the binding of STACs to SIRT1 remain elusive, due to the difficulty in obtaining a detailed X-ray crystallographic structure of the full-length enzyme. To address this, we developed an engineered hSIRT1 (mini-hSIRT1) that is biochemically equivalent to the full-length enzyme with respect to basal catalytic activity and activation by STACs. X-ray crystallographic analysis of mini-hSIRT1 resulted in the first detailed structural determination of a fully functional human SIRT1 with a bound small molecule activator. The details of STAC binding to mini-hSIRT1 were translated to the full-length enzyme using structure-guided mutagenesis which corroborated the importance of key amino acids in the binding of STACs. These data are important in elucidating the molecular basis for STAC-mediated activation of hSIRT1 which will be critical for the development of future therapeutic agents.

## Results

### Mini-hSIRT1 design and characterization

**Figure 1:**
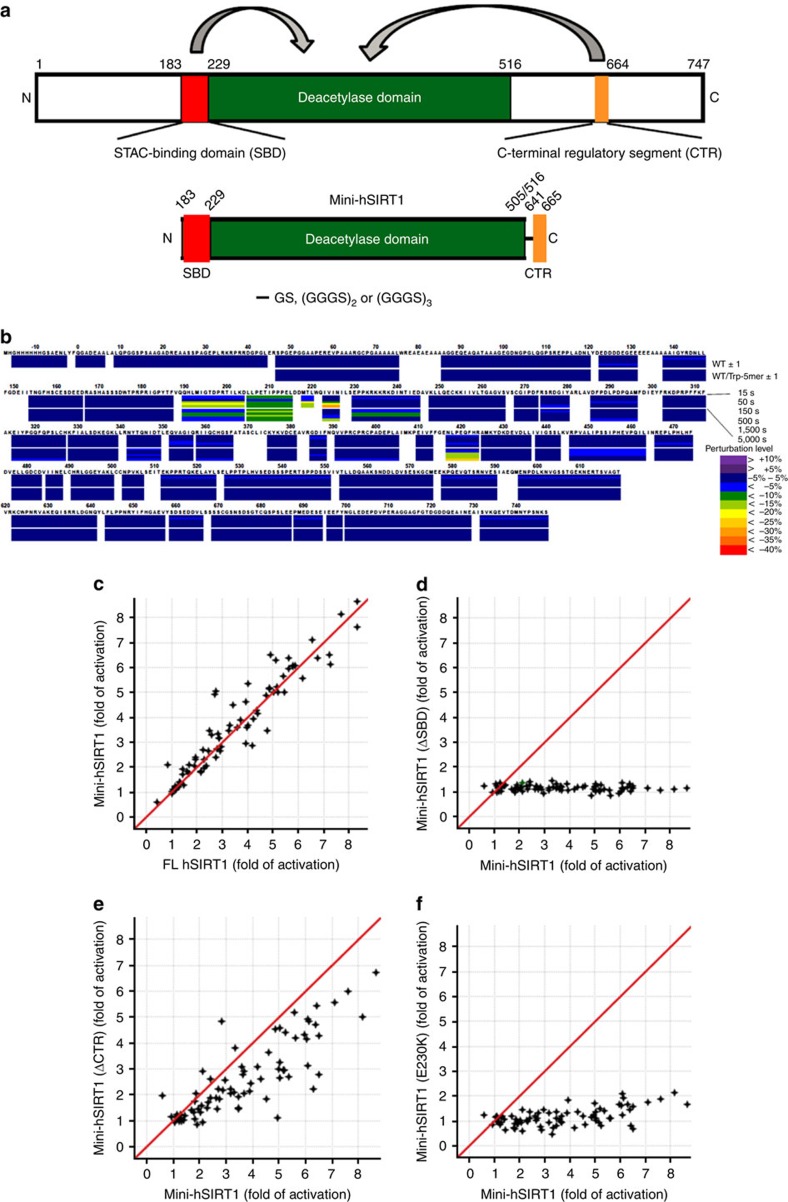
Mini-hSIRT1 construct design and characterization. (**a**) Schematic diagram of human full-length hSIRT1 and Mini-hSIRT1 constructs. The N-terminal SBD, the central catalytic domain and the CTR are highlighted in red, green and orange. (**b**) Heat map of the HDX-MS perturbation of binding of **1** to hSIRT1 in the absence or presence of Ac-p53(W5) (Trp-5mer) at six different time points (15–5000, s). (**c**) Pivot plot of the activation by a chemically diverse STAC set using the Ac-p53(W5) substrate for mini-hSIRT1 versus full-length hSIRT1, as measured by OAcADPR assay. The red line represents *y*=*x* correlation. (**d**) Pivot plot of the STAC activation of mini-hSIRT1(ΔSBD) versus mini-hSIRT1. (**e**) Pivot plot the STAC activation of mini-hSIRT1(ΔCTR) versus mini-hSIRT1. (**f**) Pivot plot of the STAC activation of mini-hSIRT1(E230K) versus mini-hSIRT1.

To identify and characterize the key functional regions of hSIRT1, we performed hydrogen-deuterium exchange mass spectrometry (HDX-MS) on the full-length hSIRT1 protein. The rate of H–D exchange is highly dependent on the dynamic properties of the protein, with faster exchange occurring at solvent exposed and/or flexible regions and slower exchange occurring at the more buried and/or rigid regions^22^. Consistent with the previous study on hSIRT1(19–747)^16^, full-length hSIRT1 contains three major structured regions: the catalytic core region; residues 229–516 (referred to as hSIRT1cd hereafter)^3^,^20^; the N-terminal region of 183–229 immediately preceding the catalytic core and a remote region following the catalytic core around 641–665, previously reported as human C-terminal regulatory segment (CTR) peptide^18^; and murine essential for SIRT1 activity peptide^23^. (Fig. 1a and Supplementary Fig. 1a).

To probe the STAC-binding site on hSIRT1, HDX-MS was performed in the absence or presence of STAC **1** (Supplementary Fig. 2). Addition of **1** reduces the H–D exchange rate around residues 183–229 in the N-terminal region of hSIRT1, suggesting that this domain is involved in STAC binding. Hereafter, this domain is referred to as the STAC-binding domain (SBD) (Fig. 1a,b and Supplementary Fig. 1a). Addition of **1** to hSIRT1 in the presence of a p53-derived peptide substrate (Ac-p53(W5))^13^ results in perturbation of the H–D exchange rates around the SBD and further protection at the substrate-binding site (residues 417–424) in the catalytic domain compared with the hSIRT1/Ac-p53(W5) complex, indicating that STAC binding in the N-terminal domain and substrate binding within the catalytic domain of hSIRT1 are coupled (Fig. 1b). This is consistent with the previous observation that STACs enhance substrate binding to hSIRT1, thereby increasing hSIRT1 catalytic efficiency^12^.

In the absence of the CTR peptide, the catalytic core (hSIRT1cd) only shows ∼15% of the activity of the full-length enzyme using deacetylation assay conditions previously reported (Supplementary Fig. 1b)^13^. The addition of the CTR peptide restores the catalytic activity of hSIRT1cd, to 80% of that of full-length hSIRT1, consistent with previous observations^23^,^24^ (Supplementary Fig. 1b). Kinetic characterization reveals that the CTR peptide restores activity by lowering the *K*_M_ values for both peptide substrate and NAD^+^ of hSIRT1cd by 4–5-fold (Supplementary Table 1).

Taken together, the above data suggest a tripartite architecture for a minimally functional hSIRT1 that includes: (1) the central domain constituting the basic catalytic machinery; (2) the N-terminal SBD that mediates STAC binding and activation; and (3) the C-terminal CTR peptide which stabilizes the catalytic domain resulting in more efficient deacetylase activity. Based on this, we designed several hSIRT1 constructs encompassing all three of the minimal structural elements covalently bound, which we termed mini-hSIRT1s. The constructs span 183–505 or 183–516, which are connected to the CTR peptide via a flexible poly-glycine/serine linker (GS, (GGGS)_2_ or (GGGS)_3_) (Fig. 1a)^25^. The *K*_M_ and *k*_cat_ values are comparable between mini-hSIRT1 constructs and the full-length enzyme, as are the IC_50_ values for the hSIRT1 inhibitors EX-527 (ref. 26) or NAM, confirming functional fidelity of mini-hSIRT1s (Supplementary Tables 2 and 3). In addition, there is an excellent correlation between mini-hSIRT1 and the full-length enzyme with respect to STAC-mediated activation across a broad set of chemotypes (Fig. 1c). Removal of the SBD completely abolishes STAC-mediated activation of mini-hSIRT1, confirming the critical importance of this domain for activation (Fig. 1d). In contrast, mini-hSIRT1 lacking the CTR retains a significant level of STAC activation (Fig. 1e) suggesting that CTR is not required for STAC-mediated activation. Finally, the E230K mutation also attenuates STAC-mediated activation in mini-hSIRT1 as in the full-length enzyme^16^ (Fig. 1f). Collectively, these observations demonstrate that at half the molecular size, mini-hSIRT1 can serve as an active and activatable surrogate for full-length hSIRT1.

### Structure of the mini-hSIRT1-STAC complex

Although the X-ray crystallographic structures of the hSIRT1 catalytic domain and complex of SIRT1cd/CTR have been reported^17^,^18^, no structure of the full-length enzyme is available. Solving the structure of the full-length hSIRT1 has been challenging, likely due to the conformational flexibility of the extended N- and C-terminal domains^16^,^27^. The mini-hSIRT1 constructs, which contain only the functionally critical regions of the N and C-terminal domains, afforded us the opportunity to crystallize a functional surrogate of the full-length enzyme. We successfully crystallized mini-hSIRT1 (183–505-(GGGS)2-CTR) with STAC **1** used in the HDX-MS experiments and determined the structure of the complex (mini-hSIRT1/**1**) at 3.1 Å by molecular replacement using a search model based on the homologous model of SIRT3 (ref. 20).

**Figure 2:**
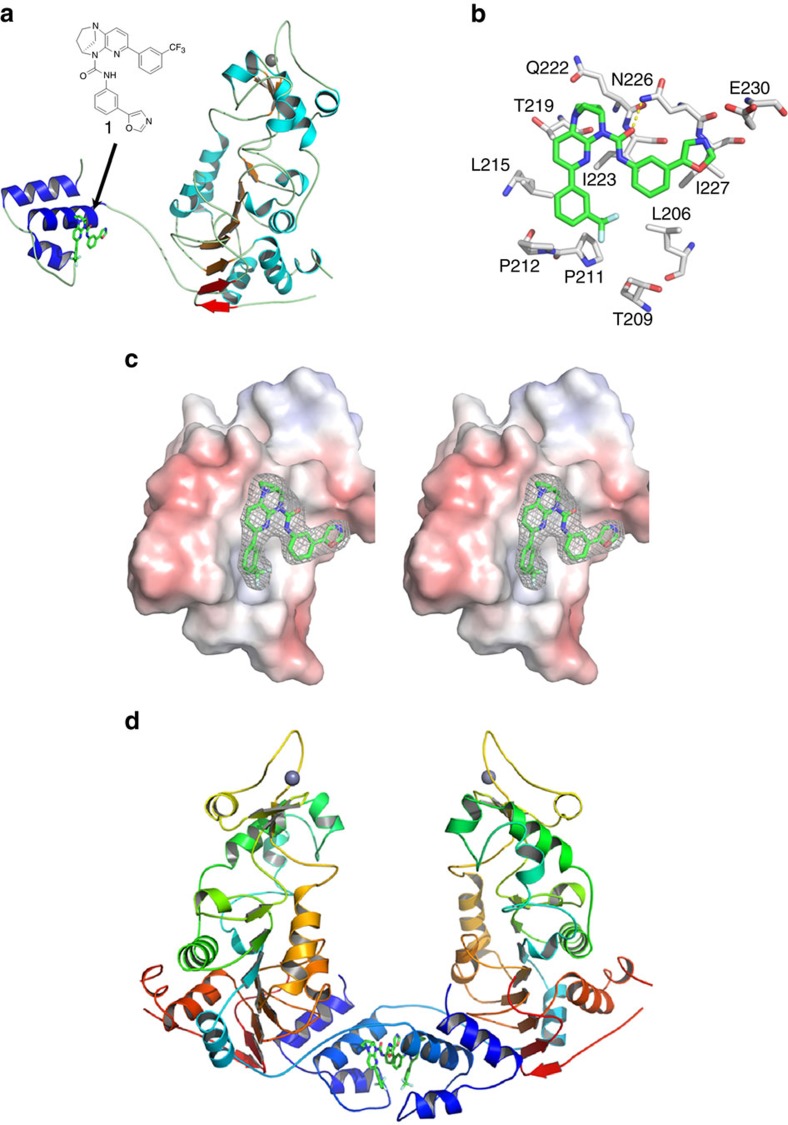
Structure of Mini-hSIRT1/1 complex. (**a**) Structure of Mini-hSIRT1/**1** complex shown in ribbon diagram. The α-helices and β-strands in the catalytic domain are shown in cyan and orange, respectively. The α-helices in the N-terminal SBD are shown in blue. The β-strands in the C-terminal CTR are shown in red. All the loops are shown in light gray. The STAC **1** is shown in green, red and blue for carbon, oxygen and nitrogen atoms. The zinc ion is shown as a gray sphere. (**b**) hSIRT1-binding site of **1** with interacting residues shown in stick representation. Hydrogen bonds are shown as yellow dotted lines. (**c**) Stereo view of the electrostatic surface potential of the N-terminal SBD and electron density map of STAC **1**. The electrostatic potential is contoured at the 5 kT/e level, with red denoting negative potential and blue denoting positive potential. The 2Fo-Fc omit map of **1** is contoured at 1.0 σ level. (**d**) Crystallographic dimer of Mini-hSIRT1/**1** complex. The protein ribbon is rainbow-colored from blue at the N-terminus to red at the C-terminus.

Mini-hSIRT1 comprises a catalytic domain that assumes a Rossmann-fold large lobe and a zinc-binding small lobe common to all sirtuins^19^, an N-terminal three-helical bundle encompassing the SBD and a C-terminal β-hairpin CTR peptide^18^ (Fig. 2a). The CTR peptide mediates β-augmentation with the six-stranded β-sheet of the Rossmann-fold lobe of the catalytic domain (Fig. 2a), in agreement with the HDX-MS results of hSIRT1cd perturbation upon CTR binding (Supplementary Fig. 3a,b). The CTR-mediated β-augmentation appears to stabilize the active site of the hSIRT1cd which restores the *K*_M_ values observed for both acetylated peptide and NAD^+^ substrates^18^. The N-terminal SBD forms an independently folded, three-helix bundle with **1** binding to the helix-turn-helix (H2-T-H3) motif within the SBD, consistent with the HDX-MS and enzyme kinetic results (Fig. 2a). STAC **1** interacts extensively with the hydrophobic side chains of Leu^206^, Thr^209^ (methyl), Pro^211^, Pro^212^, Leu^215^, Thr^219^ (methyl), Ile^223^ and Ile^227^ from the H2-T-H3 motif with only one hydrophilic interaction: a hydrogen bond with Asn^226^ (Fig. 2b). The major mini-hSIRT1/**1**-binding site is a shallow hydrophobic surface depression with an off-center, deeper hydrophobic pocket, that the CF_3_ group of **1** occupies (Fig. 2c). This is consistent with the observed structure-activity relationships developed across multiple STAC chemotypes, indicating the requirement of overall flatness of the core scaffold maintained by an intramolecular hydrogen bond^28^. A remarkable similarity in terms of domain configuration is observed between the mini-hSIRT1 structure and that of yeast Sir2 with both having an N-terminal helical bundle and the C-terminal β-augmentation by a β-hairpin beyond the typical Rossmann-fold large lobe^29^ (Supplementary Fig. 3c). However, yeast Sir2 does not include the 130 amino acid insertion (510–640) observed in hSIRT1 and appears to be a natural ‘mini-SIRT1' in yeast.

Interestingly, a STAC-mediated dimer of mini-hSIRT1 related by crystallographic symmetry was observed in the crystal lattices (Fig. 2d). Size exclusion chromatography (SEC) indicates that the apparent size of mini-hSIRT1 increases in the presence of STAC **1**, which is likely correspondent to the mini-hSIRT1 dimer species (Supplementary Fig. 4). We are currently attempting to determine if the observed crystallographic dimer has any relevance in the observed biology of STAC-mediated SIRT1 activation.

**Figure 3:**
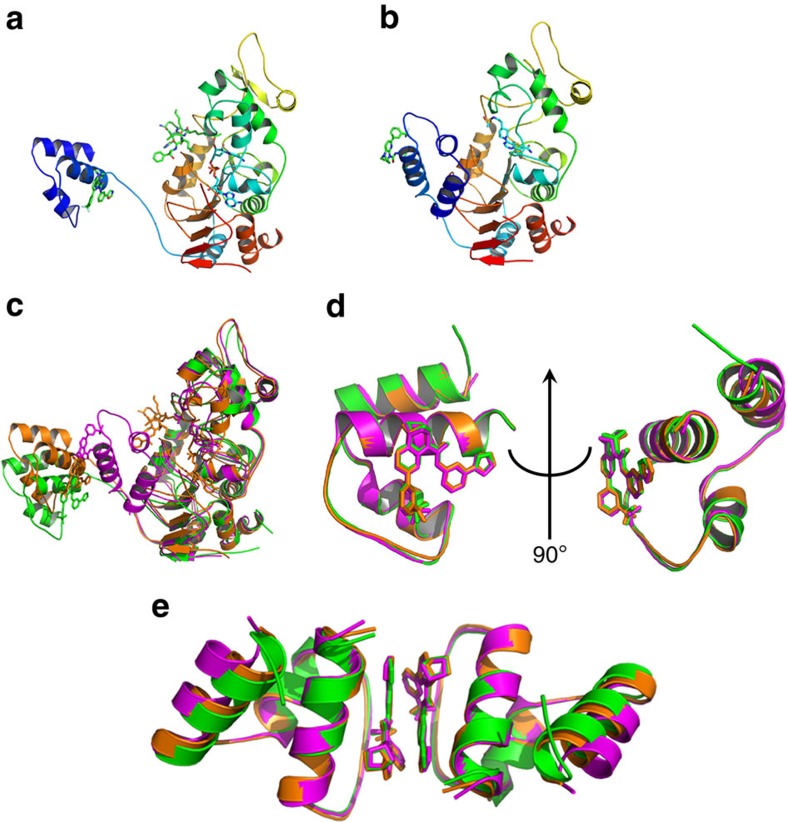
Structures of Mini-hSIRT1-STAC/ligand complex. (**a**) Structure of mini-hSIRT1/**1**/Ac-p53 7-mer/CarbaNAD quaternary complex. The STAC **1** and Ac-p53 7-mer are shown in green, red and blue for carbon, oxygen and nitrogen atoms. The CarbaNAD is shown in cyan, red, blue and orange for carbon, oxygen, nitrogen and phosphate atoms. The protein ribbon is rainbow-colored from blue at the N-terminus to red at the C-terminus. (**b**) Structure of mini-hSIRT1/**1**/**2** complex. The mini-hSIRT1 shown in this complex is hSIRT1(183–516)-GS-CTR as the same complex containing hSIRT1(183–505)-(GGGS)_2_-CTR diffracts to 3.5 Å even though the structures are almost identical. The STAC **1** is shown in green, red and blue for carbon, oxygen and nitrogen atoms. The Inhibitor **2** is shown in cyan, red, blue and yellow for carbon, oxygen, nitrogen and sulfur atoms. The protein ribbon is rainbow-colored from blue at the N-terminus to red at the C-terminus. (**c**) Structural comparison of mini-hSIRT1/**1** complex (green), mini-hSIRT1/**1**/Ac-p53 7-mer/CarbaNAD quaternary complex (orange) and mini-hSIRT1/**1**/**2** complex (magenta). (**d**) Superimposition of the SBD domains from mini-hSIRT1/**1** complex (green), mini-hSIRT1/**1**/Ac-p53 7-mer/CarbaNAD quaternary complex (orange) and mini-hSIRT1/**1**/**2** complex (magenta). (**e**) Comparison of the STAC-mediated dimer interface of mini-hSIRT1/**1** complex (green), mini-hSIRT1/**1**/Ac-p53 7-mer/CarbaNAD quaternary complex (orange) and mini-hSIRT1/**1**/**2** complex (magenta).

In addition to the mini-hSIRT1/**1** complex structure, we also determined a 2.73 Å structure of a quaternary complex of mini-hSIRT1, **1**, a seven amino acid peptide substrate derived from p53 (Ac-p53), and the nonhydrolyzable NAD^+^ analog carbaNAD and a 2.74 Å structure mini-hSIRT1/**1** in complex with an active-site directed inhibitor **2** (ref. 30) that occupies the peptide and NAD^+^-binding sites (Fig. 3a,b and Supplementary Fig. 2). In the quaternary complex structure, the Ac-p53 peptide and carbaNAD bind to the active site cleft between the large and small lobes. Ac-p53 adopts an extended conformation, similar to the backbone-mediated β-strand like interactions observed in many Sirtuin/Ac-peptide complexes^19^,^31^. The main chain amide groups of Ac-p53 form hydrogen bonds with those of the residues Gly^415^ and Glu^416^ from the small lobe and those of the residues Lys^444^ and Arg^446^ from the large lobe (Supplementary Fig. 5a). The hydrogen bonds between the amide of the peptide +1 position and that of Arg^446^ render a potential interaction between the side chain and a bulky and hydrophobic +1 residue, which might be important in STAC-mediated hSIRT1 activation^13^. The acetyl-lysine side chain inserts into a hydrophobic cavity lined by Phe^414^, Leu^418^ and Val^445^. The acetyl group is sandwiched between His^363^ and Phe^297^, with the ɛ-N of the acetyl-lysine hydrogen bonded with the carbonyl oxygen of Val^412^, which maintains the orientation and the extended conformation of the acetyl-lysine side chain. CarbaNAD also makes multipoint contacts with hSIRT1 (Supplementary Fig. 5b), most of which are similar to those observed in reported Sirtuin/NAD^+^ strucutres^19^,^31^. Inhibitor **2** occupies both the acetyl-lysine-binding site and the NAM-binding C-pocket of mini-hSIRT1, similar to the recently reported structure of the SIRT3/**2** complex^30^ (Supplementary Fig. 5c). Similar to SIRT3, binding of substrates or the active-site inhibitor leads to domain closure, bringing the small and large lobes together^20^,^21^,^30^. Comparison of the three structures shows that the locations of the N-terminal SBD domain relative to the catalytic core are divergent among the three structures, likely to be impacted by different crystal packings (Fig. 3c). The hinge residue, Arg^234^, is located within the polybasic linker (residues 233–238, KRKKRK) and anchors the N-terminal SBD to the catalytic domain through a salt bridge formed between its guanidinium group and the carboxylate group of Asp^475^ and hydrogen bonds to the carbonyl groups of His^473^ and Val^459^ (Supplementary Fig. 5d). In contrast, the SBD domain itself is relatively rigid, with a superimposable STAC-binding helix-turn-helix (H2-T-H3) motif with only the first helix tilting out slightly in the mini-hSIRT1/**1**/**2** complex structure (Fig. 3d). The STAC-mediated dimer interface also seems to be conserved among the three structures (Fig. 3e).

### Site-directed mutagenesis of the STAC binding pocket

We used site-directed mutagenesis on the full-length hSIRT1 to confirm the key residues of the SBD that were identified by the mini-hSIRT1 structures. The following point mutants of full-length hSIRT1 were generated probing three classes of residues: (a) residues which appear to directly interact with STACs (T219A, I223A, N226A and I227A); (b) SBD residues with no apparent role in activator binding (Q222A and V224A); and (c) Glu^230^, previously demonstrated to be important for SIRT1 activation^16^ (E230K, E230A and E230Q) (Fig. 2b). None of the mutants significantly impaired the basal catalytic activity using the Ac-p53(W5) substrate or affected inhibition by EX-527, a Trifluoroacetic acid (TFA)-p53 peptide (Ac-RHK-K^TFA^-L-Nle-F-NH_2_), or NAM (Supplementary Tables 4 and 5).

**Figure 4:**
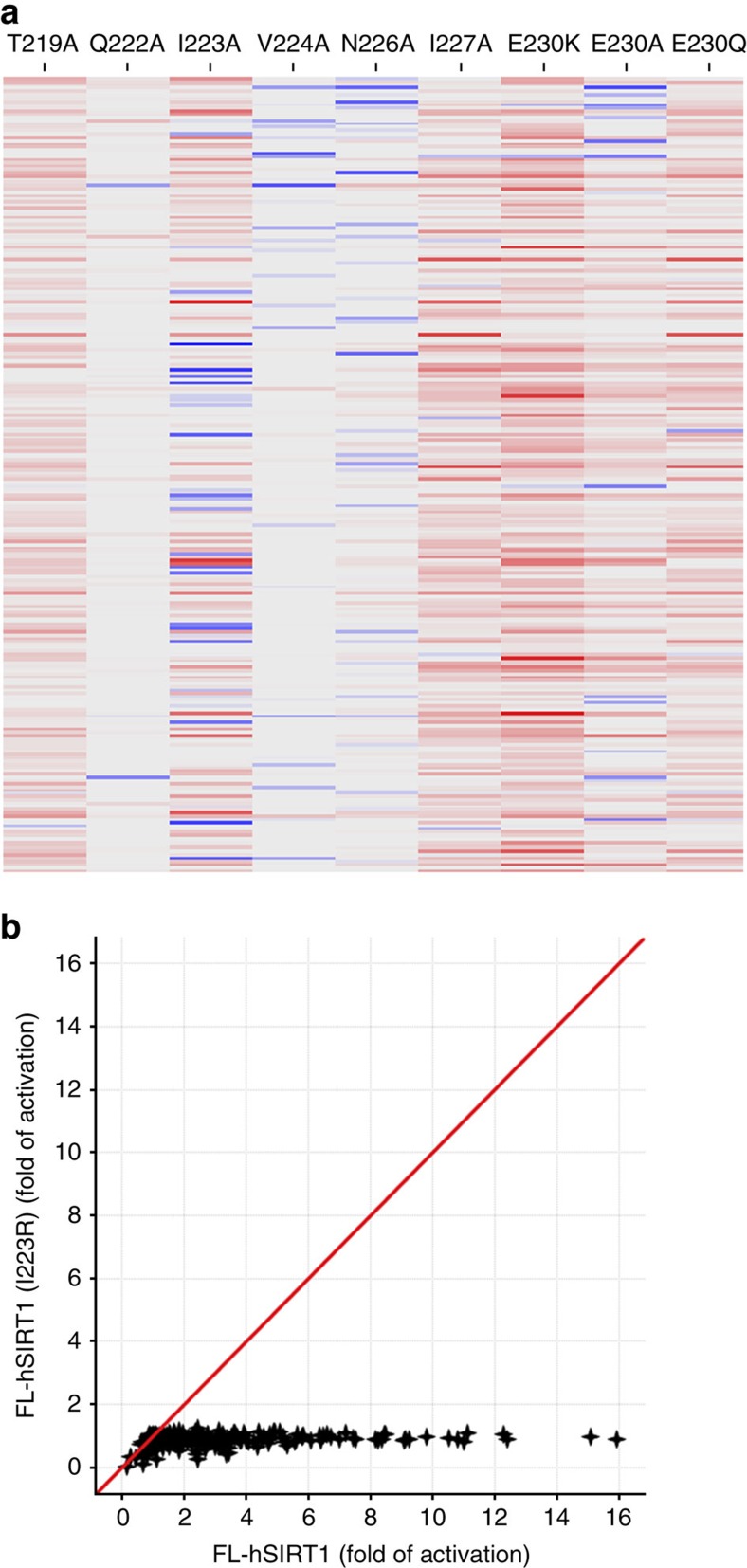
Activation of full-length hSIRT1 mutants. (**a**) Heat map of the ratio of wild-type/mutant fold-activation for hSIRT1 mutants for each compound from a structurally diverse collection of 246 STACs. Ratios from 0.80 to 1.16 are colored gray. This range covers one s.d. of the mean for V224A (0.98±0.18-fold, Supplementary Fig. 5b) which does not affect activation. Activation impairment denoted as a red gradient (ratios of 1.17/6.78). Activation enhancement is shown as a blue gradient (ratios of 0.78/0.24). (**b**) Comparison of the fold-activation of I223R versus wild-type hSIRT1 with a structurally diverse collection of STACs. All of the data were generated with the OAADPr assay using the Ac-p53(W5) substrate. A different compound set (∼250 STACs) was used for the site-directed mutagenesis studies compared with that used for the initial characterization of mini-hSIRT1 (∼80 STACs).

The impact of the mutations on activation was first assessed by comparing the fold-activation of wild-type versus mutant full-length SIRT1 using a structurally diverse set of 246 STACs tested at a fixed concentration of 25 μM. Additionally, we investigated the effect of the mutations on STAC binding versus activation by monitoring shifts in their EC_50_ and maximum activation values respectively using a panel of eight compounds (STACs **1, 3–9**, Supplementary Fig. 2, Supplementary Tables 6 and 7). T219A, I223A and I227A all exhibit broad impairment of activation with increases in EC_50_ values compared with wild-type hSIRT1, suggesting impaired activator binding consistent with the mini-hSIRT1 structures (Fig. 4a and Supplementary Table 6). Interestingly, I223A was the most compound-dependent mutant, exhibiting both attenuated and enhanced activation, the latter particularly for STACs containing an ortho-CF_3_ substituted phenyl ring (Fig. 4a and Supplementary Fig. 6a). In the crystal structure, Ile^223^ lies directly beneath the STAC and lines the pocket into which the meta-CF_3_ of **1** inserts. The cavity created by mutation of Ile^223^ to Ala would be expected to better accommodate an ortho- versus a meta-substitution. This observation further validates the key molecular interactions governing STAC binding indicated in the structure and points to strategies for altering STAC interaction with the SBD.

Asn^226^ appears to form a hydrogen bond between its carboxamide nitrogen and the carbonyl oxygen of **1** on the surface of the protein (Fig. 2b). However, activation of N226A was only minimally impaired compared with the wild type (Fig. 4a). The small contribution from this H-bond is likely because of its high solvent exposure.

In contrast to the above mutants, Q222A and V224A displayed normal activation which is consistent with their positions away from the STAC in the mini-hSIRT1/**1** structure (Fig. 4a and Supplementary Fig. 6b,c). Importantly, all of these data obtained with full-length hSIRT1 are consistent with what the mini-SIRT1 crystal structures predict further validating the biochemical significance of these structures.

Despite the broad impact of the mutations described above, none of them completely abolished activation of hSIRT1 as seen with removal of the SBD. As Ile^223^ lies directly beneath the bound STAC and activation of I223A is highly compound-dependent, we reasoned that further mutating this residue, to incorporate a more disruptive interaction in hSIRT1, would result in a more highly activation-impaired full-length enzyme. To test this hypothesis, we prepared an I223R mutant to introduce steric bulk and charge into the hydrophobic STAC-binding site. Consistent with our hypothesis, activation is completely lost for all 246 activators using both the Ac-p53(W5) or FOXO-3a substrate peptides (Fig. 4b and Supplementary Fig. 6d), while the basal catalytic activity and inhibition by EX-527, TFA-p53 peptide or NAM is not impacted in the I223R mutant (Supplementary Tables 4, 5 and 8). SEC of the STAC-binding deficient mini-hSIRT1 I223R mutant remains the same in the presence of STAC **1**, confirming that the observed mini-hSIRT1 dimerization in solution is mediated by STAC **1**. (Supplementary Fig. 4).

Mutation of Glu^230^ to either Lys or Ala has been recently reported to broadly impair activation by STACs, although the mechanism by which this occurs is unclear^16^. We tested activation of E230K, E230A and E230Q full-length hSIRT1 proteins and found that the maximum activation is impaired with a minimal impact on the EC_50_ (Supplementary Tables 6 and 7), suggesting a role for Glu^230^ in the formation or stabilization of the activated conformation of hSIRT1. Activation of E230Q is also broadly impaired indicating that the negative charge of Glu^230^ is important for stabilizing the activated conformation of hSIRT1 and likely interacts with a positively charged residue in the activated state.

### Allosteric coupling between STAC and substrate binding

**Figure 5:**
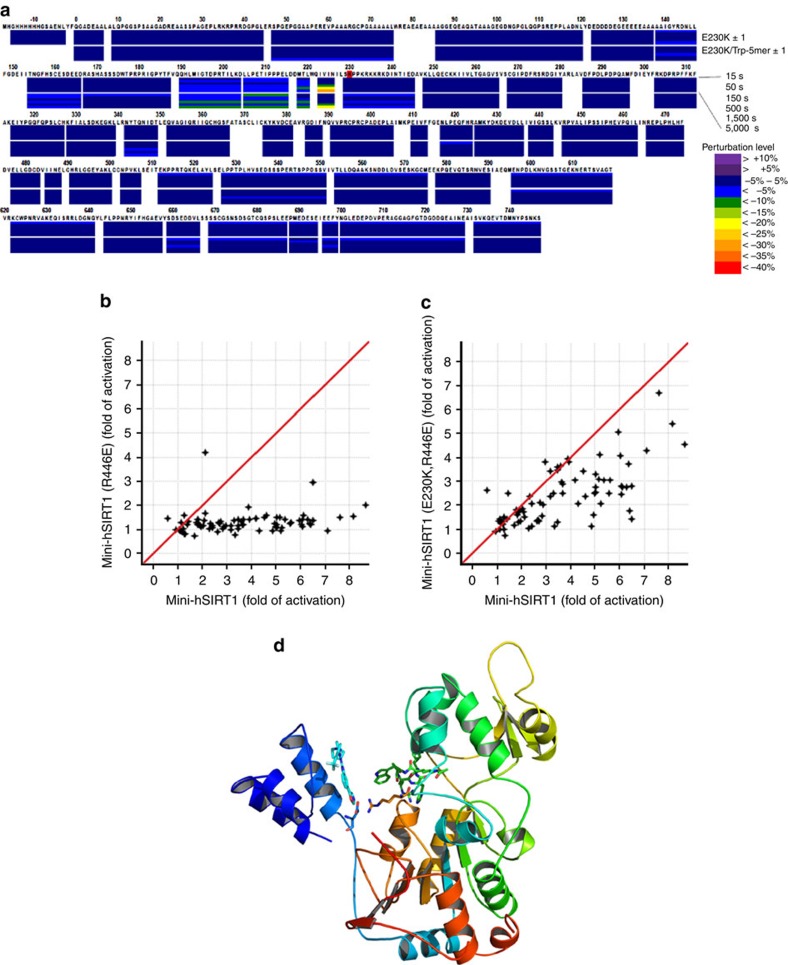
E230K impairs the coupling between STAC and substrate binding and potential role of an electrostatic interaction between Glu^230^ and Arg^446^ in the activated conformation. (**a**) Heat map of the HDX-MS perturbation of binding of **1** to hSIRT1(E230K) in the absence or presence of Ac-p53(W5) at six different time points (15–5000, s). (**b**) Pivot plot of the STAC activation of mini-hSIRT1(R446E) versus mini-hSIRT1. (**c**) Pivot plot of the STAC activation of the double charge-reversal mutant mini-hSIRT1(R446E/E230K) versus mini-hSIRT1. (**d**) Speculative model of the activated conformation of SIRT1. Glu^230^ and Arg^446^ are shown in stick representation. The protein ribbon is rainbow-colored from blue at the N-terminus to red at the C-terminus. **1** and modeled Ac-p53(W5) are shown in cyan and green, respectively.

We further probed the role of Glu^230^ in STAC-mediated activation using HDX-MS which revealed that, in contrast to wild-type hSIRT1, STAC binding to the E230K mutant no longer confers protection around the peptide-binding site in the E230K/**1**/Ac-p53(W5) complex (Fig. 5a and Supplementary Fig. 7). This indicates that the E230K mutation may negatively affect the coupling between the STAC and substrate-binding sites. The HDX-MS and activation data together suggest that Glu^230^ is not directly involved in STAC binding but is instead, a critical residue mediating the coupling of STAC and substrate binding to promote activation.

The observation that regions outside STAC-binding site and substrate-binding site show minimal perturbation in HDX-MS in the presence of both ligands suggests the possibility that the two binding sites might be physically close to each other in the activated conformation. Given this observation and the importance of the negative charge of Glu^230^ for activation, we postulated that Arg^446^ located at the active site might be a possible electrostatic partner for Glu^230^, stabilizing the activated conformation of hSIRT1 and mediating the observed coupling. To this end, we made the mini-hSIRT1 R446E/E230K double mutant with E230K and R446E mini-hSIRT1 as controls. Mini-hSIRT1(E230K) mutant does not affect the basal catalytic activity using the Ac-p53(W5) substrate, as observed in full-length SIRT1 (Supplementary Table 2). Both mini-hSIRT1(R446E) mutant and mini-hSIRT1(E230K,R446E) mutant show higher *K*_M_ values for both peptide substrate and NAD^+^, which might result from the potential hydrophobic interaction between the aliphatic part of Arg^446^ side chain and the substrate as R446F mutant does not affect the basal catalytic activity (Supplementary Table 2). Whereas either E230K or R446E results in significant attenuation of STAC activation of mini-hSIRT1, the E230K,R446E double mutant partially restores STAC-mediated activation of mini-hSIRT1 compared with E230K or R446E, supporting the importance of potential electrostatic interaction between Glu^230^ and Arg^446^ in the activated conformation (Fig. 5b,c).

## Discussion

In this study, we describe the design and construction of a functional mini-hSIRT1 that recapitulates three key features of full-length hSIRT1: (1) the steady-state enzyme kinetics and inhibition; (2) the STAC activation profile across multiple chemotypes; and (3) STAC activation impairment by E230K mutation. We used this mini-hSIRT1 construct to obtain the first reported crystallographic structure of hSIRT1 with a bound STAC. The biochemical and structural characterization confirms the hSIRT1 intramolecular interactions between the CTR and catalytic domain, which enhance the basal deacetylation activity of hSIRT1. The structures of the mini-hSIRT1-STAC complex reveal the detailed architecture of the STAC-binding site, which was validated in full-length hSIRT1 by site-directed mutagenesis. The STAC-binding site appears to be a shallow hydrophobic surface depression, which matches the flat and hydrophobic nature of the STACs. Consequently, the mini-hSIRT1-STAC structure reported here provides important information for future structure-based drug design. In addition, we demonstrated the coupling between the STAC-binding site and the active site using HDX-MS, which is impaired by the previously reported E230K mutant. Structure-based mutagenesis suggested that the electrostatic interaction between Glu^230^ and Arg^446^ might stabilize the activated conformation. The exact nature of the activated conformation is still elusive. Apparently, Glu^230^ and Arg^446^ in the reported structure are too far to make electrostatic interactions. Modeling with the N-terminal SBD treated as a rigid body to rotate around the hinge point Arg^234^ suggests that rotating the SBD around Arg^234^ could bring Glu^230^ close to Arg^446^, which interestingly also bring the STAC **1** close to the active site, esp. the hydrophobic side chain of the modeled Ac-p53(W5) (Fig. 5d). This model is highly speculative and needs to be tested experimentally, but does seem to be attractive as it might help to explain the requirement of some hydrophobic moiety on the peptide for SIRT1 activation by STACs, by participating in the composite activator-binding site and facilitating the formation of the activated conformation. However, some key questions remain to be answered with further investigation: (1) is the observed STAC induced SIRT1 dimer relevant for SIRT1 activation by STACs? (2) How does the STAC binding in the N-terminal SBD enhance the substrate binding at the active site, which is not obvious from the comparison of current structures of Mini-SIRT1/**1** and Mini-SIRT1/**1**/ p53 7-mer/CarbaNAD? (3) Does STAC binding itself induce conformational change of the N-terminal SBD, in other words, what does apo Mini-SIRT1 look like? The current structures served as a stepping stone to answer these important questions and elucidate the mechanism of activation of SIRT1 by STACs. In summary, the results presented here provide unambiguous visual and functional proof of direct allosteric activation of hSIRT1 by small molecules, and provide a basis for further elucidation of the mechanism of hSIRT1 activation by STACs.

## Methods

### Protein cloning, expression and purification

Mini-hSIRT1 constructs were cloned into a modified pET21b vector (Novagen). The protein was expressed in *Escherichia coli* BL21-Gold (DE3) cells (Stratagene) as an N-terminal fusion to a hexahistidine affinity tag with integrated Tobacco Etch Virus (TEV) protease site. A single colony was inoculated in LB media containing 100 μg ml^−1^ ampicillin at 37 °C, 250 r.p.m. until the A_600_ reached 0.3. The culture was then transferred to 16 °C, 250 r.p.m. until the A_600_ reached 0.6. Isopropyl 1-thio-β-D-galactopyranoside was added to a final concentration of 0.2 mM, and expression was continued at 16 °C, 250 r.p.m. overnight. Cells were collected by centrifugation, and the pellet was resuspended in lysis buffer (25 mM HEPES, pH 7.5, 200 mM NaCl, 5% glycerol and 5 mM 2-mercaptoethanol) and sonicated to break the cells. Supernatant was separated from cell debris by centrifugation at 10,000*g* for 40 min at 4 °C and loaded onto a Ni-NTA column (Qiagen) that equilibrated with the buffer containing 25 mM HEPES, pH 7.5, 200 mM NaCl, 5% glycerol, 5 mM 2-mercaptoethanol and 20 mM imidazole. The column was washed with five column volumes of the buffer containing 25 mM HEPES, pH 7.5, 200 mM NaCl, 5% glycerol, 5 mM 2-mercaptoethanol and 50 mM imidazole, and eluted with the buffer containing 25 mM HEPES, pH 7.5, 200 mM NaCl, 5% glycerol, 5 mM 2-mercaptoethanol and 250 mM imidazole. The eluted protein was dialyzed in lysis buffer and digested with TEV protease (Invitrogen) to remove the N-terminal His tag at 4 °C overnight. The protein was loaded on a second Ni-NTA column equilibrated with lysis buffer. The untagged protein was eluted by the buffer containing 25 mM HEPES, pH 7.5, 200 mM NaCl, 5% glycerol, 5 mM 2-mercaptoethanol and 5 mM imidazole. The purified protein was dialyzed against the dialyzing buffer containing 20 mM Tris-HCl, pH 8.0, 250 mM NaCl, 5% glycerol and 10 mM dithiothreitol, and concentrated. The protein was further purified by a S200 column (GE Healthcare) to 95% purity as assessed by SDS–polyacrylamide gel electrophoresis analysis stained by Coomassie Brilliant Blue R-250 and concentrated to 10–15 mg ml^−1^ in the dialyzing buffer.

Full-length human SIRT1 (hSIRT1) proteins were expressed with a N-terminal His_6_ tag and purified as described in Hubbard *et al.*^16^ except for Q222A, and I223R SIRT1 which were purified using an ÄKTAxpress (GE Lifesciences). Each cell paste was resuspended in buffer A (50 mM Tris-HCl pH 7.5, 250 mM NaCl, 25 mM imidazole and 0.1 mM TCEP) with 1,000 U Benzonase nuclease (Sigma-Aldrich, St Louis, MO, USA) supplemented with cOmplete, EDTA-free Protease Inhibitor Cocktail Tablets (Roche) on ice. Cells were disrupted by pulse sonication with 50% on and 50% off for 12 min total at 40 W. Insoluble debris was removed by centrifugation. Clarified supernatant was directly loaded onto a 1 ml HisTrap FF Crude column (GE Lifesciences). After washing with buffer A, SIRT1 was eluted with buffer B (50 mM Tris-HCl pH 7.5, 250 mM NaCl, 500 mM imidazole and 0.1 mM TCEP). Protein was further purified by SEC in buffer C (50 mM Tris-HCl pH 7.5, 300 mM NaCl and 0.1 mM TCEP) using a Hi-load Superdex 200 16/60 column (GE Lifesciences). Enzyme concentrations were determined by Bradford assay using bovine serum albumin (BSA) as a standard. Final protein purity was assessed by gel densitometry. Proteins were confirmed by liquid chromatography/mass spectrometry. All proteins were greater than 90% pure except V224A and T219A (80%) and E230A (85%).

### SIRT1 deacetylation reactions

SIRT1 deacetylation reactions were performed in reaction buffer (50 mM HEPES-NaOH, pH 7.5, 150 mM NaCl, 1 mM dithiothreitol and 1% dimethylsulfoxide (DMSO)) at 25 °C monitoring either NAM production using the continuous PNC1/GDH coupled assay^32^ or *O*-acetyl ADP ribose (OAcADPr) production by mass spectrometry^16^. Final concentrations of the PNC1/GDH coupling system components used were 20 units per ml bovine GDH (Sigma-Aldrich), 1 μM yeast PNC1, 3.4 mM α-ketoglutarate and 220 μM NADH or NADPH. An extinction coefficient of 6.22 mM^−1^cm^−1^ and a pathlength of 0.81 cm were used to convert the absorbance at 340 nm to product concentration for the 150 μl reactions used. Assays monitoring OAcADPr production were performed in reaction buffer with 0.05% BSA and time points were taken by quenching the deacetylation reaction with a stop solution which gave a final concentration of 1% formic acid and 5 mM NAM. Quenched reactions were diluted fivefold with 1:1 acetonitrile:methanol and spun at 5,000*g* for 10 min to precipitate protein before being analyzed with an Agilent RapidFire 200 High-Throughput Mass Spectrometry System (Agilent, Wakefield, MA) coupled to an ABSciex API 4000 mass spectrometer fitted with an electrospray ionization source. The p53-based Ac-p53(W5) (Ac-RHKKAcW-NH2) and FOXO-3a 21-mer (Ac-SADDSPSQLSKAcWPGSPTSRSS-NH2) peptides were obtained from Biopeptide. Deacetylation assays used the Ac-p53(W5) substrate unless otherwise noted.

Substrate *K*_M_ determinations were performed by varying one substrate concentration at a fixed, saturating concentration of the second substrate. SIRT1 activation and inhibition assays were run in reaction buffer with 0.05% BSA at 25 °C and analyzed using the OAcADPr assay. Enzyme and compound were preincubated for 20 min before addition of substrates. For the activation screen of full-length hSIRT1, a structurally diverse set of 246 compounds was tested in duplicate at a final concentration of 25 μM each. In order to be sensitive to *K*_M_-modulating activators, substrate concentrations of approximately one-tenth their *K*_M_ values were used. The dose-dependence of eight compounds was tested and the fold-activation data were described by equation (1)


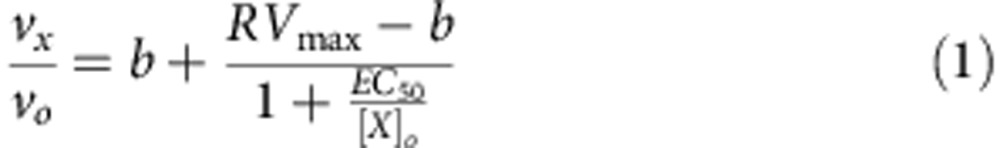


where *v*_*x*_/*v*_*0*_ is the ratio of the reaction rate in the presence (*v*_*x*_) versus absence (*v*_*0*_) of activator (*X*), *RV*_max_ is the relative velocity at infinite activator concentration, *EC*_50_ is the concentration of activator required to produce one-half RV_max_ and b is the minimum value of *v*_*x*_/*v*_*0*_.

### SEC assay

The assays were performed with a Superdex 75 10/300 GL column (GE healthcare) injecting 100 μl samples containing 10 μM mini-hSIRT1 in the absence or presence of 100 μM STAC, dissolved in 50 mM HEPES-NaOH, pH 7.5, 150 mM NaCl and 0.5 mM TCEP. Binding reactions were incubated for 1 h at room temperature before injection into the column.

### HDX-MS

On-exchange experiment of SIRT1. H/D-exchange reactions followed by pepsin digestion, desalting, high-performance liquid chromatographic separation and mass spectrometric analysis were carried out using a fully automated system, described in detail elsewhere^22^. Particular to this set of experiments, on-exchange reactions were initiated by mixing 20 μl of a SIRT1 stock solution (0.77 mg ml^−1^ SIRT1,±3.88 mM Ac-p53(W5),±192 μM ligand, in 1.9% DMSO) and 20 μl of 100 mM phosphate, pH read 7.0 in D2O. The 50% D_2_O mixture was incubated at 0 °C for 15, 50, 150, 500, 1,500 or 5,000 s. For SIRT1 (229–516), on-exchange reactions were initiated by mixing 4 μl of a SIRT1 stock solution (1.36 mg ml^−1^ SIRT1 (229–516),±1.67 mM CTR peptide) and 36 μl of 200 mM phosphate, pH read 7.0 in D2O. The 90% D2O mixture was incubated at 0 °C for 15, 50, 150, 500, 1,500, or 5,000 s. Addition of 20 μl of 1.6 M guanidine hydrochloride (GuHCl), 0.8% formic acid, pH 2.3, quenched the on-exchange reaction immediately prior to being analyzed.

General protein process for standard HDX sample. The quenched solution was passed through a pepsin column (104 μl bed volume) filled with porcine pepsin (Sigma) immobilized on Poros 20 AL media (Life Technologies, Carlsbad, CA, USA) per the manufacturer's instructions, with 0.05% aqueous TFA (200 μl min^−1^) for 2 min. The digested fragments were temporarily collected onto a reverse phase trap column (4 μl bed volume) and desalted. The peptide fragments were then eluted from the trap column and separated by a C18 column (BioBacis-18; Thermo Scientific, San Jose, CA, USA) with a linear gradient of 13% solvent B to 40% solvent B over 23 min (solvent A, 0.05% TFA in water; solvent B, 95% acetonitrile, 5% buffer A; flow rate 10 μl min^−1^). Mass spectrometric analyses were carried out using a LTQ OrbiTrap XL mass spectrometer (Thermo Fisher Scientific) with capillary temperature at 200 °C.

Digestion/separation optimization and nondeuterated experiment of SIRT1. Before H/D-exchange experiment, digestion and separation conditions were optimized to yield high sequence coverage of SIRT1 by peptic fragments with high resolution under nondeuterated conditions. In this step, a mixture of 20 μl of 0.77 mg ml^−1^ (9.2 μM) SIRT1 and 20 μl of H2O was quenched by the addition of 20 μl of various acidic buffers. For SIRT1 (229–516), a mixture of 4 μl of a SIRT1 stock solution (1.36 mg ml^−1^ SIRT1 (229–516) and ±1.67 mM CTR peptide) and 36 μl of H2O was quenched by the addition of 20 μl of various acidic buffers. The quenched mixtures were subjected to aforementioned general protein process. The nondeuterated peptic fragments were identified by Sequest in Proteome Discoverer 1.1 (Thermo Fisher Scientific).

Fully deuterated experiment of SIRT1. The fully deuterated sample was prepared by incubating a mixture of 45 μl of 0.77 mg ml^−1^ (9.2 μM) SIRT1 with 45 μl of 100 mM TCEP in D2O, pH 2.5 at 60 °C for 3 h. For SIRT1 (229–516), the fully deuterated sample was prepared by incubating a mixture of 9 μl of 1.36 mg ml^−1^ (41.7 μM) SIRT1 (229–516) with 81 μl of 100 mM TCEP in D2O, pH 2.5 at 60 °C for 3 h. After incubation, the sample was kept at 0 °C before being quenched identically to an on-exchanged solution and subjected to the general protein process.

Determination of deuteration level of each peptide after on-exchange reaction.

The centroids of peptide isotopic envelopes were measured using the in-house-program developed in collaboration with Sierra Analytics (Modesto, CA, USA). Corrections for back-exchange during the protein processing step were made employing the following standard equation equation (2):


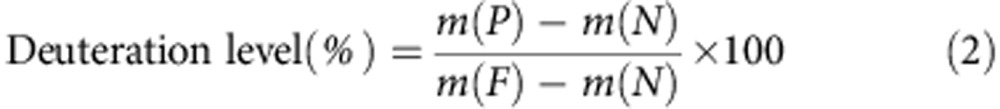


where *m*(*P*), *m*(*N*) and *m*(*F*) are the centroid value of partially deuterated (on-exchanged) peptide, nondeuterated peptide and fully deuterated peptide, respectively.

### Protein crystallization, data collection and structure determination

The crystals of mini-hSIRT1/**1** binary complex were obtained by hanging drop vapor diffusion method at 18 °C. The crystals appeared overnight and grew to a final size of ∼0.1 × 0.1 × 0.1 mm within 2 days. 10 mg ml^−1^ protein was incubated with compound **1** for ∼1 h and the molar ratio of compound **1**:protein is 5:1 with 1% DMSO. The drop was composed of 1 μl of protein/compound mixture and 1 μl crystallization buffer of 0.2 M Magnesium chloride, 0.1 M Tris pH 8.5, and 16% w/v PEG 4000. The crystals of mini-hSIRT1/**1**/**2** were obtained by hanging drop vapor diffusion method at 18 °C. The crystals appeared overnight and grew to a final size of∼0.1 × 0.1 × 0.1 mm within 2 days. 10 mg ml^−1^ protein was incubated with compound **1** for about 1 h, then incubated with compound **2** for 2 h and the molar ratio of compound **1**:compound **2**:protein is 5:5:1 with 2% DMSO. The drop was composed of 1 μl of protein/compound mixture and 1 μl crystallization buffer of 0.55 M Sodium chloride, 0.1 M MES pH 6.5 and 20% w/v PEG 4000. The crystals of mini-hSIRT1/**1**/p53–7mer/carbaNAD complex were obtained by hanging drop vapor diffusion method at 18 °C. The crystals appeared overnight and grew to a final size of∼0.1 × 0.1 × 0.1 mm within 2 days. 10 mg/ml protein was incubated with compound **1** for about 1 h, then incubated with p53-7mer and CarbaNAD for 2 h and the molar ratio of compound **1**:p53-7mer: CarbaNAD:protein is 5:5:10:1 with 1% DMSO. The drop was composed of 1 μl of the protein/compound/substrate mixture and 1 μl of the crystallization buffer of 5% v/v Tacsimate, pH.00.1 M HEPES pH 7.0 and 10% w/v PEG 5000 MME.

The crystals were cryo-protected in mother liquor containing 20% glycerol before being flash-frozen in liquid nitrogen. Diffraction data were collected at SSRF BL17U1, APS 21-ID-D or APS 21-ID-G beamlines at 100 K and processed using the Xia2 program^33^. The molecular replacement software Phaser^34^ was used to solve the structure with a search model containing residues 242–494 based on the homolog model of SIRT3 (PDB code: 3GLU) initially and later also with a search model of SIRT1 (PDB code 4IG9) when available. Iterative structure refinement and model building were performed between Phenix.refine^35^ and Coot^36^. Bulk solvent correction and Translation/Libration/Screw-motion (TLS) refinement were used during the refinement and model building. Detailed information regarding the diffraction data, refinement and structure statistics is listed in Table 1.

Details of the chemical compounds synthesismethods are provided in Supplementary Methods.

## Additional information

**How to cite this article:** Dai, H. *et al.* Crystallographic structure of a small molecule SIRT1 activator-enzyme complex. *Nat. Commun.* 6:7645 doi: 10.1038/ncomms8645 (2015).

## Supplementary Material

Supplementary Information Supplementary Figures 1-7, Supplementary Tables 1-8, Supplementary Methods and Supplementary References

## Figures and Tables

**Table 1:** Data processing and refinement statistics.

	**Mini-SIRT1/1**	**Mini-SIRT1/1/2**	**Mini-SIRT1/1/ p53 7-mer/CarbaNAD**
*Data collection*			
Resolution (Å)*	45.67–3.10 (3.18–3.10)	39.98–2.73 (2.81–2.73)	91.36–2.74 (2.81–2.74)
Space group	I2_1_2_1_2_1_	P6122	I4122
Unit-cell parameters			
a (Å)	99.19	122.15	94.51
b (Å)	111.64	122.15	94.51
c (Å)	132.52	104.92	356.84
Completemess (%)*	99.5 (99.8)	99.9 (100.0)	99.5 (99.4)
Redundancy*	4.8 (4.9)	17.6 (18.2)	9.6 (9.9)
Average I/σI*	17.4 (2.0)	38.8 (4.0)	20.7 (3.3)
Rmerge (%)*	6.7 (78.1)	5.3 (80.3)	8.2 (82.9)
			
*Refinement*			
Resolution (Å)*	45.67–3.10 (3.34–3.10)	39.98–2.73 (3.01–2.73)	45.68–2.74 (2.87–2.74)
*R*_work_ (%)*	18.7 (29.7)	19.1 (23.7)	18.3 (27.3)
*R*_free_ (%)*	23.8 (37.7)	23.5 (30.0)	22.1 (33.2)
r.m.s.d In bond lengths (Å)	0.006	0.004	0.005
r.m.s.d in bond angles (°)	1.039	0.898	0.938
Mean B factors (Å2)	103.3	92.0	71.9

^*^Values in parentheses are for the highest- resolution shell.
