# Supplementary material for: Crystallographic structure of a small molecule SIRT1 activator-enzyme complex
Source: Nat Commun. 2015 Jul 2;6:7645. doi: 10.1038/ncomms8645 (PMC4506539; doi:10.1038/ncomms8645)
Supplement: Supplementary Information — Supplementary Figures 1-7, Supplementary Tables 1-8, Supplementary Methods and Supplementary References [file ncomms8645-s1.pdf]

Supplementary Information for

Crystallographic structure of a small molecule SIRT1 activator-enzyme complex

Han Dai[1,3]*, April W. Case[1], Thomas V. Riera[1], Thomas Considine[1], Jessica E. Lee[2], Yoshitomo Hamuro[2], Huizhen Zhao[3], Yong Jiang[3], Sharon M. Sweitzer[3], Beth Pietrak[3], Benjamin Schwartz[3], Charles A. Blum[1], Jeremy S. Disch[1], Richard Caldwell[1], Bruce Szczepankiewicz[1], Christopher Oalmann[1], Pui Yee Ng[1], Brian H. White[1], Rebecca Casaubon[1], Radha Narayan[1], Karsten Koppetsch[1], Francis Bourbonais[1], Bo Wu[4], Junfeng Wang[4], Dongming Qian[5], Fan Jiang[5], Cheney Mao[5], Minghui Wang[3], Erding Hu[3], Joe C. Wu[1], Robert B. Perni[1], George P. Vlasuk[1], James L. Ellis[1,3]

[1] Sirtris, a GlaxoSmithKline company, 200 Technology Square, Suite 300, Cambridge, MA 02139, USA.

[2] ExSAR Corporation, 11 Deer Park Drive, Suite 103, Monmouth Junction, NJ 08852, USA.

[3] GlaxoSmithKline, 1250 S. Collegeville Road, Collegeville, PA 19426, USA.

[4] High Magnetic Field Laboratory, Hefei Institutes of Physical Science, Chinese Academy of Sciences, 350 Shushanhu Road, Hefei 230031, Anhui Province, China.

[5] Viva Biotech Ltd., 334 Aidisheng Rd., Zhangjiang High-tech Park, Shanghai 201203, China.

Correspondence and requests for materials should be addressed to Han Dai: Han.x.Dai@gsk.com

This Word file includes:

Supplementary Figures S1-S7
Supplementary Tables S1-S8
Supplementary Methods
Supplementary Schemes S1-S7
Supplementary References (1-2)

a

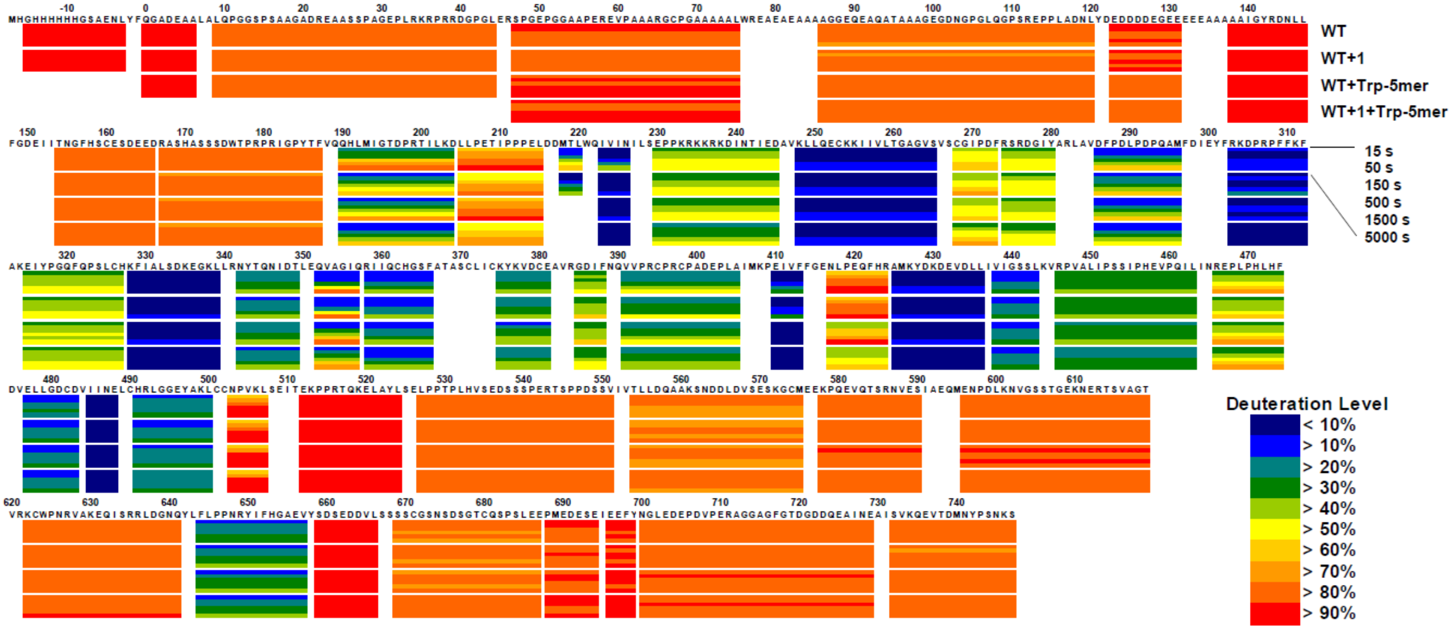

b

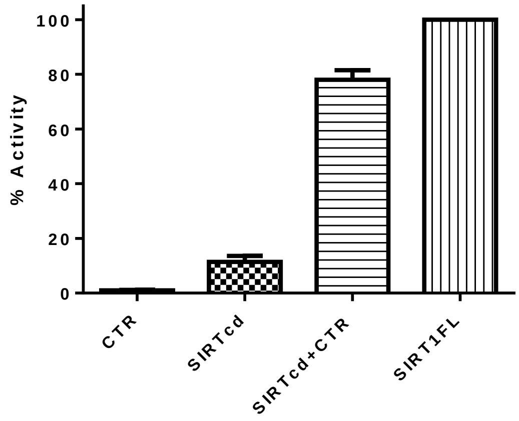

Supplementary Fig. 1. (a) HDX-MS of full length SIRT1. The warm colored regions represent high deuterium incorporation, reflecting the highly dynamic properties and the intrinsic flexibility of these regions. Conversely, the cold colored regions represent low deuterium incorporation, reflecting the rigidity of these regions. **(b)** Restoration of catalytic activity of hSIRT1cd by the addition of CTR peptide. The data were aquired using PNC1/GDH assay with 3.7 μM Ac-p53(W5) and 70 μM NAD^+.

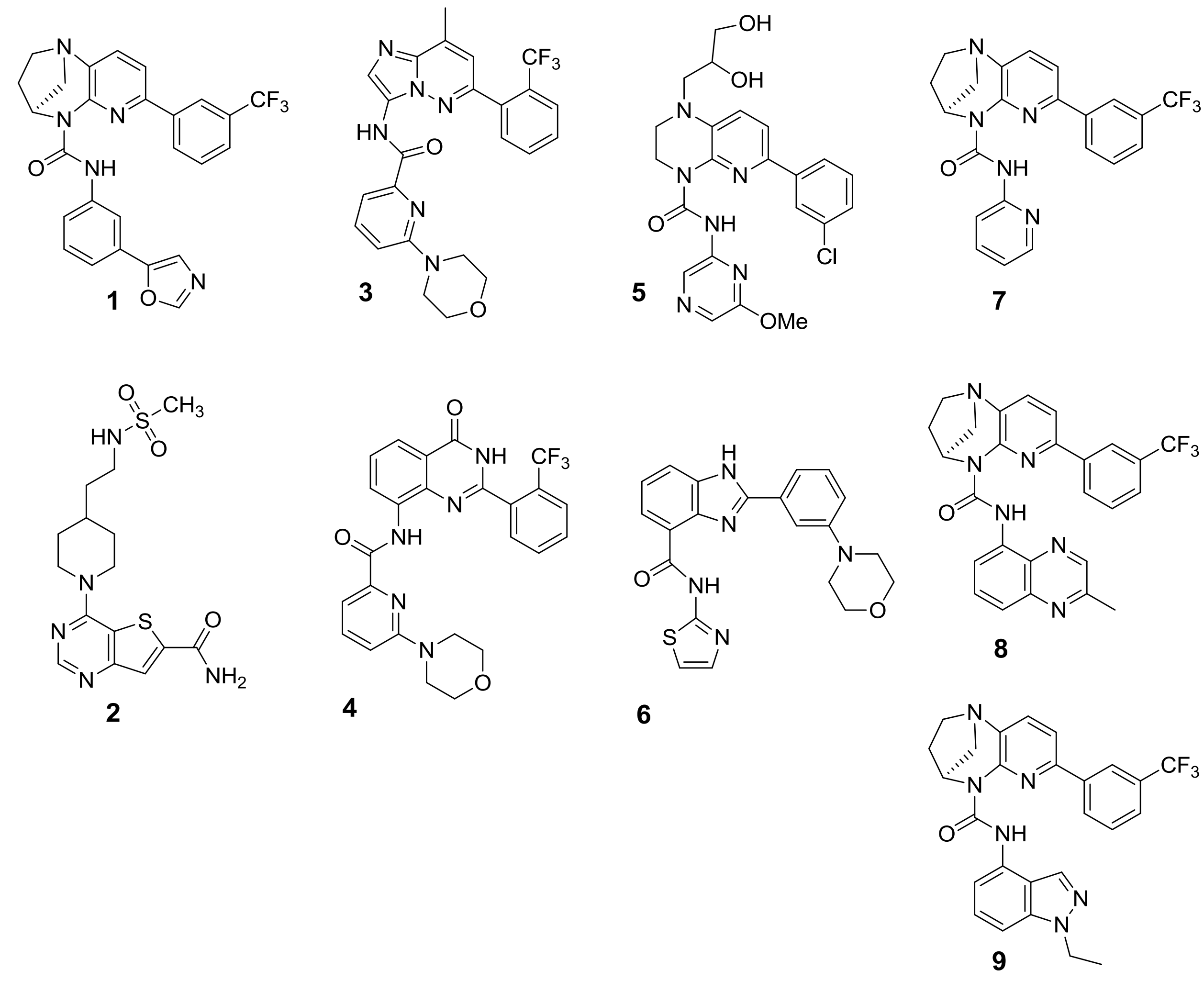

Supplementary Fig. 2. Chemical structures of synthetic SIRT1 activators (**1, 3-9**) and inhibitor (**2**).

a

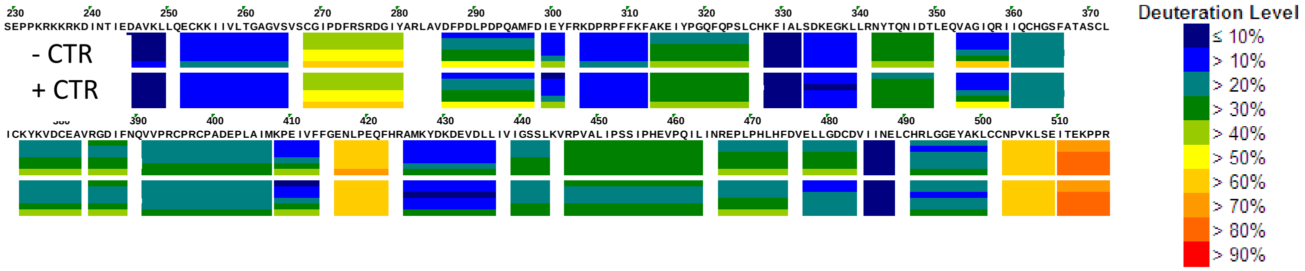
- CTR
+ CTR
Deuteration Level
≤ 10%
> 10%
> 20%
> 30%
> 40%
> 50%
> 60%
> 70%
> 80%
> 90%

b

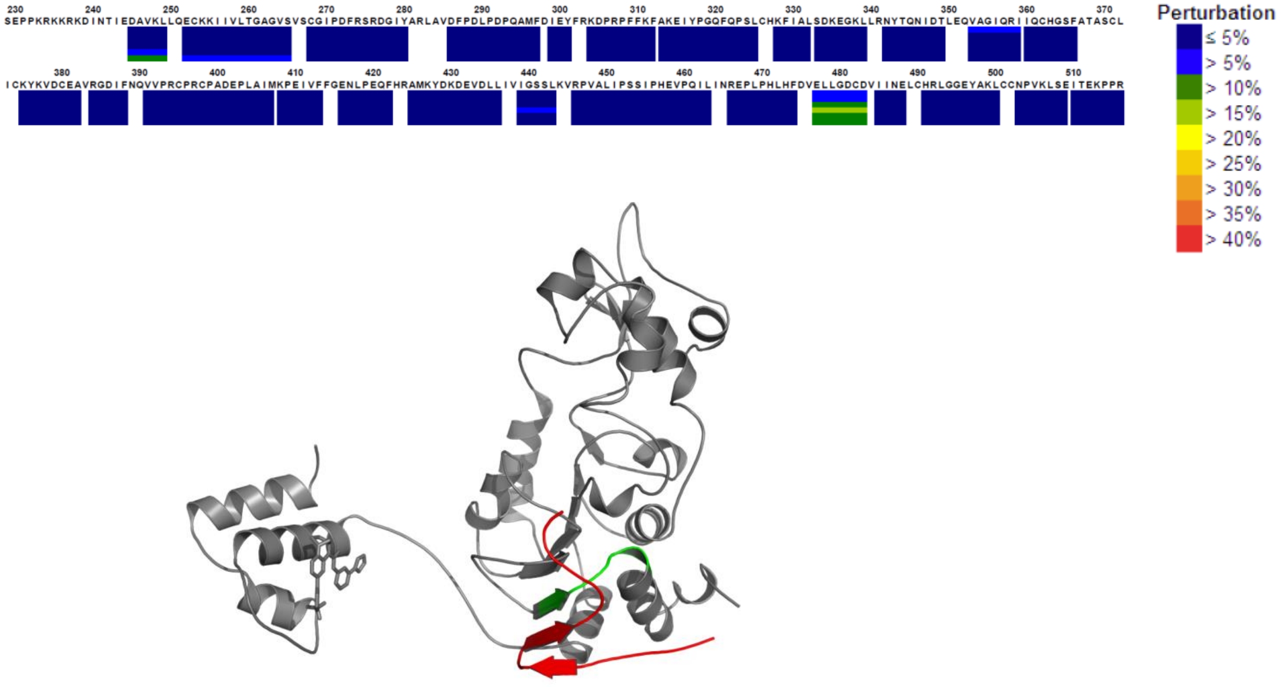
Perturbation
≤ 5%
> 5%
> 10%
> 15%
> 20%
> 25%
> 30%
> 35%
> 40%

c

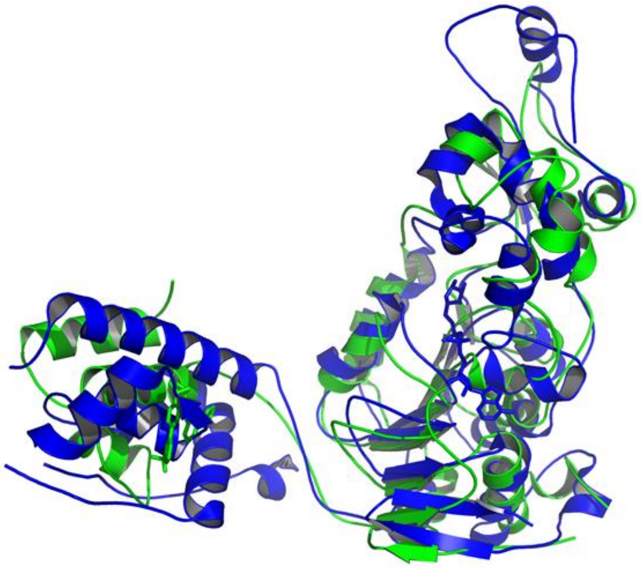

Supplementary Fig. 3. **(a)** HDX-MS profile of SIRT1cc in the absence (top) and in the presence (bottom) of CTR peptide. **(b)** Differential perturbation of the HDX-MS profile of SIRT1cc upon binding to CTR peptide. The perturbed sequence is mapped on the Mini-hSIRT1/**1** complex structure (gray) as green-highlighted region and the CTR peptide is shown in red. **(c)** Structural comparison of Mini-hSIRT1/**1** complex (Green) and ySIR2 structure in the ySIR2/ySIR4 complex (Blue).

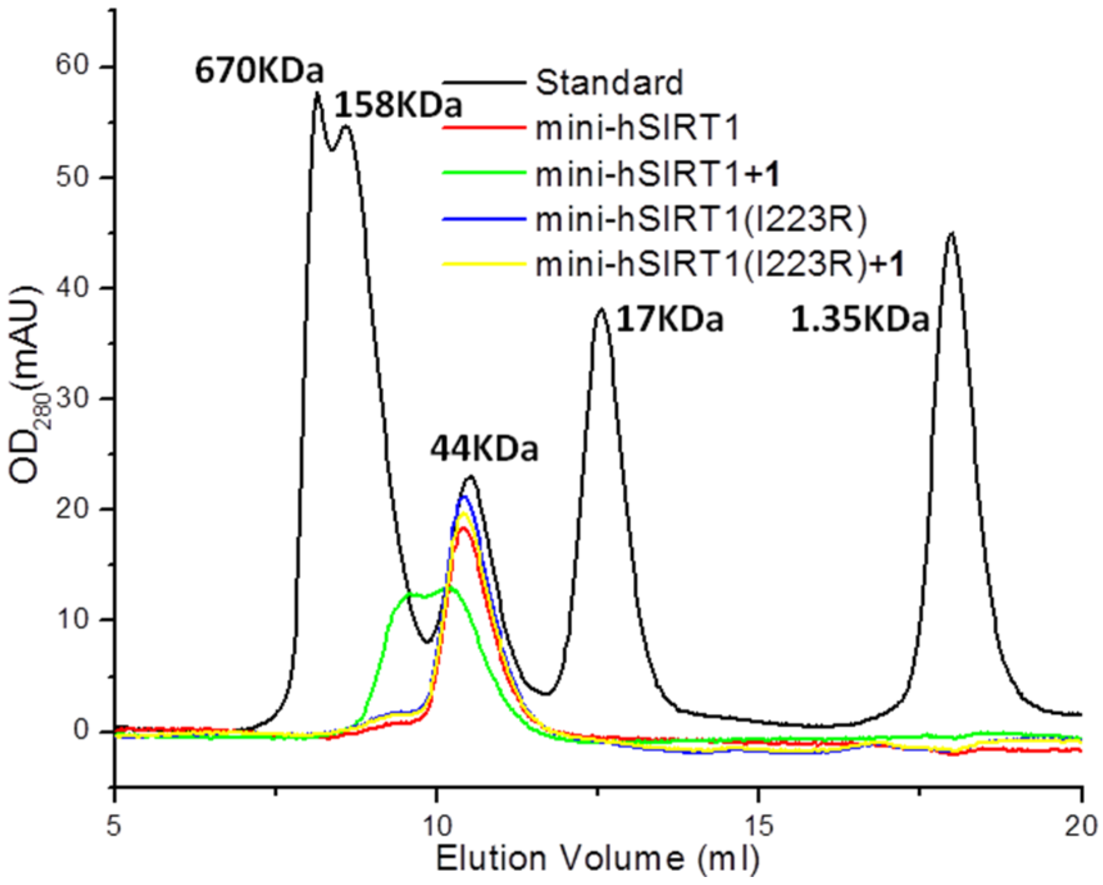

Supplementary Fig. 4. Size exclusion chromatography (SEC) of mini-hSIRT1 in the absence or presence of STAC **1**. The molecular weight standards are shown with molecular weights labeled on the top of the peaks.

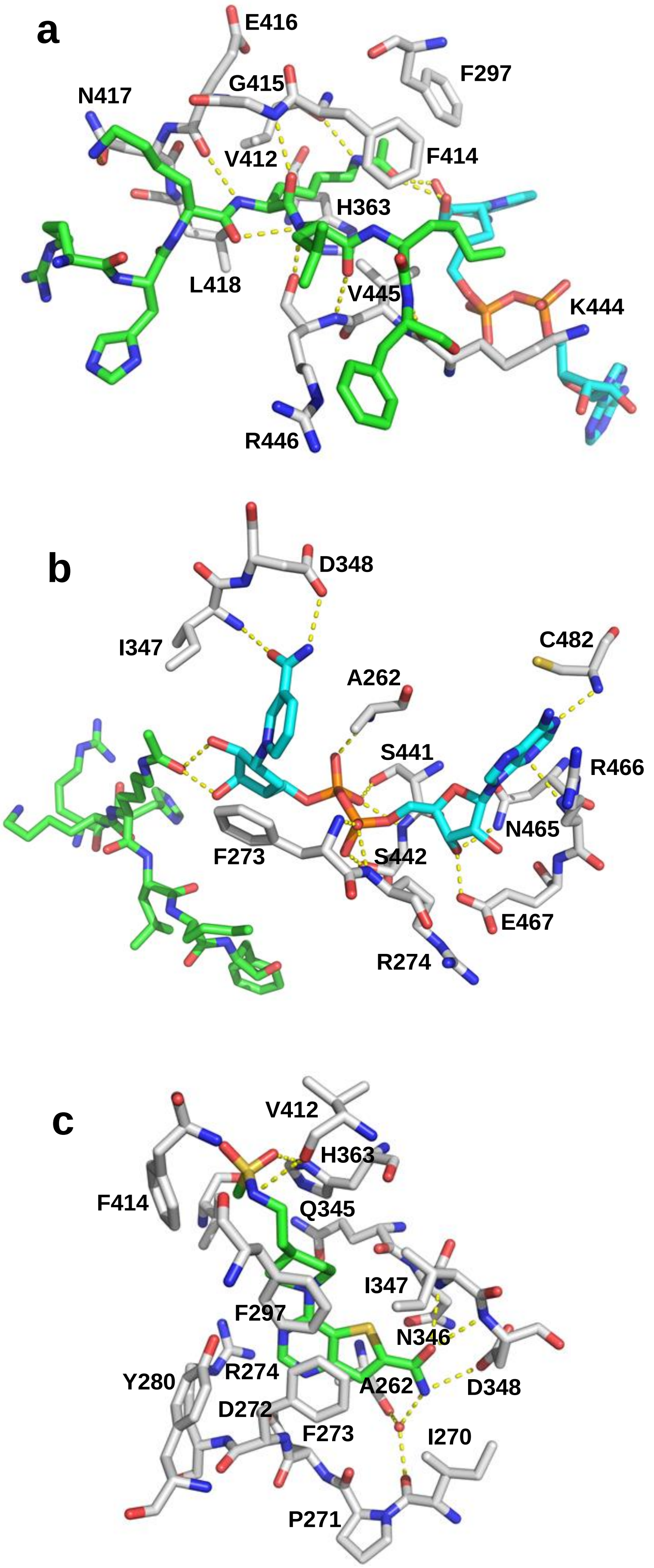

a
E416
F297
G415
N417
V412
F414
H363
L418
V445
K444
R446
b
D348
I347
C482
A262
S441
R466
F273
N465
S442
E467
R274
c
V412
H363
F414
Q345
I347
F297
N346
Y280
R274
A262
D348
D272
F273
I270
P271

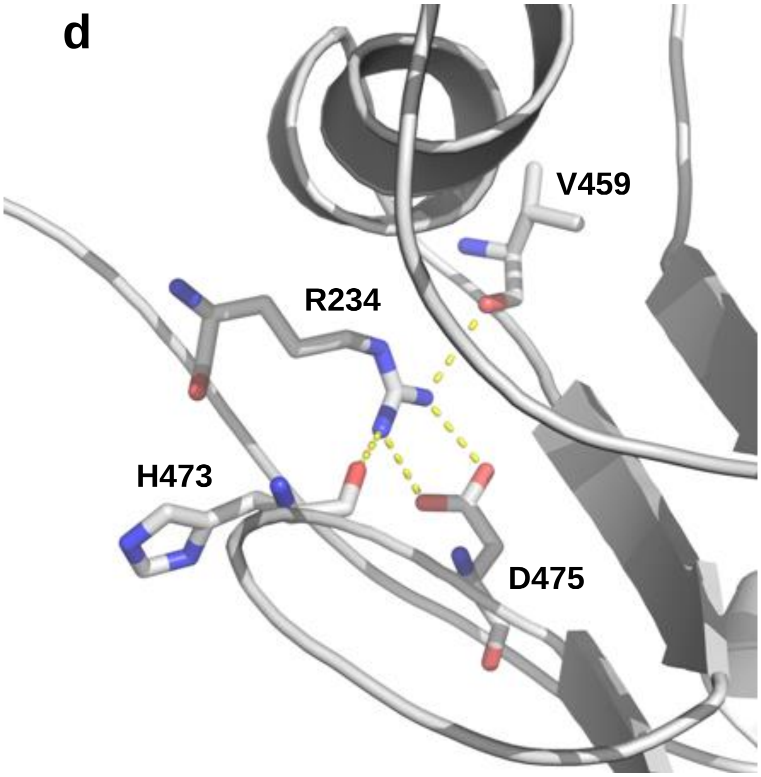

Supplementary Fig. 5. **(a)** Interface of Mini-hSIRT1/Ac-p53 7-mer interaction. The protein is shown in gray, red and blue for carbon, oxygen and nitrogen atoms. The Ac-p53 7-mer is shown in green, red and blue for carbon, oxygen and nitrogen atoms. The CarbaNAD is shown in cyan, red, blue and orange for carbon, oxygen, nitrogen and phosphate atoms. **(b)** Interface of Mini-hSIRT1/carbaNAD interaction. The protein is shown in gray, red and blue for carbon, oxygen and nitrogen atoms. The Ac-p53 7-mer is shown in green, red and blue for carbon, oxygen and nitrogen atoms. The CarbaNAD is shown in cyan, red, blue and orange for carbon, oxygen, nitrogen and phosphate atoms. **(c)** Interface of Mini-hSIRT1/**2** interaction. The protein is shown in gray, red and blue for carbon, oxygen and nitrogen atoms. **2** is shown in green, red, blue and yellow for carbon, oxygen, nitrogen and sulfur atoms. **(d)** Interactions between Arg^{234} and Asp^{475}, His^{473}, Val^{459}.

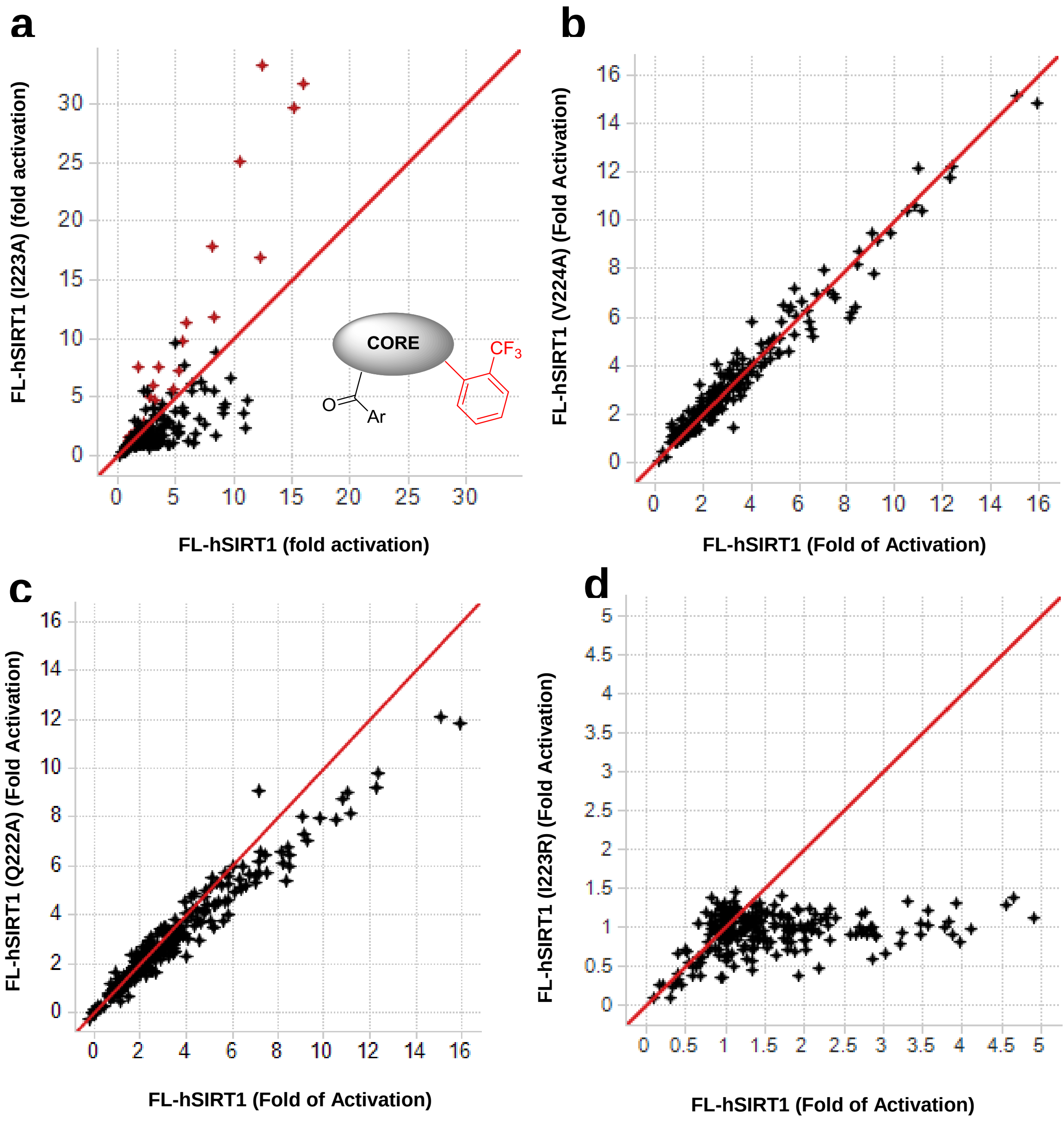

Supplementary Fig. 6. Activation comparison of wild-type versus mutant full-length hSIRT1. **(a)** Fold-activation of I223A vs wild-type (WT) hSIRT1 using the Ac-p53(W5) peptide. Red colored markers denote I223A-selective activators with the ortho-CF_3 benzyl substituent pictured. **(b)** Fold-activation of V224A vs wild-type (WT) SIRT1 using the Ac-p53(W5) peptide. **(c)** Fold-activation of Q222A vs wild-type (WT) SIRT1 using the Ac-p53(W5) peptide. **(d)** Fold-activation of I223R vs wild-type (WT) SIRT1 using the FOXO-3a 21-mer substrate. All were tested against a 246-compound set (25 μM final) using the OAcADPr assay. A y=x reference line is shown in red.

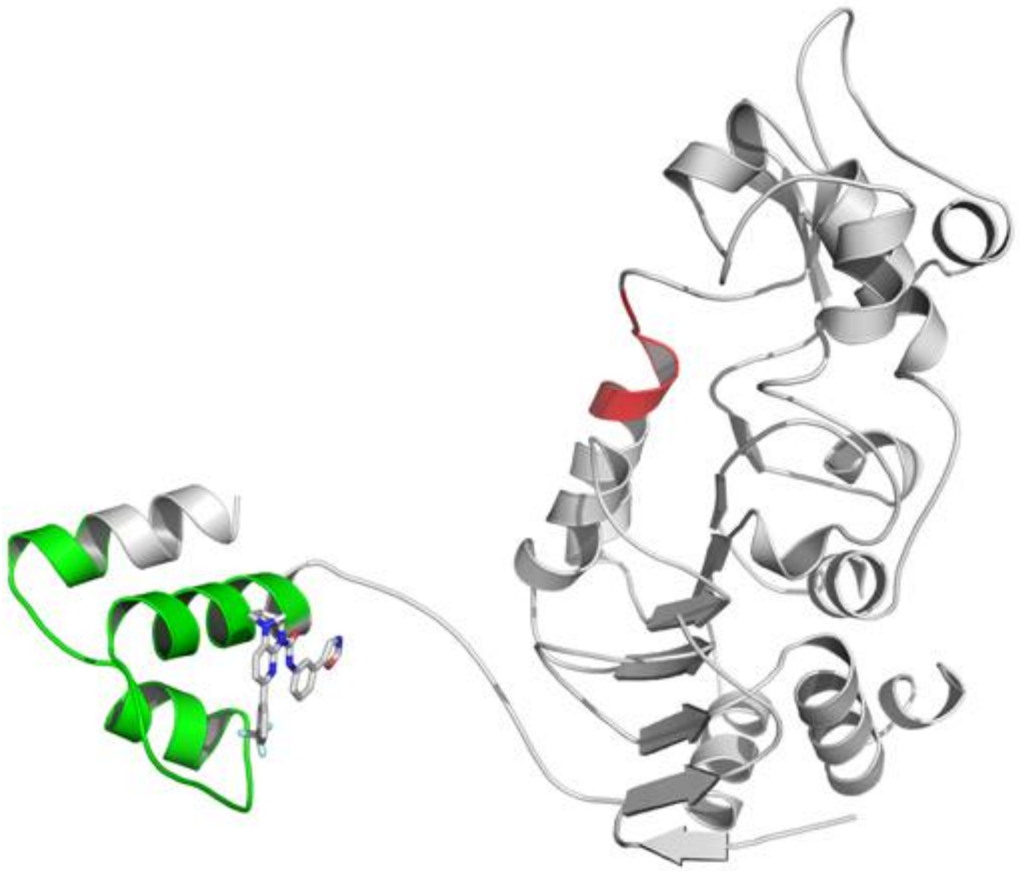

Supplementary Fig. 7. Differential perturbation of the HDX-MS profile of SIRT1 upon binding to STAC **1** in the absence or presence of Ac-p53(W5) (Trp-5mer). The perturbed sequence is mapped on the Mini-hSIRT1/**1** complex structure (gray). The green-highlighted region is pertubated on SIRT1 WT or E230K mutant upon binding to STAC **1** in the absence or presence of Ac-p53(W5) (Trp-5mer). The red-highlighted region is only pertubated on SIRT1 WT upon binding to STAC **1** in the presence of Ac-p53(W5) (Trp-5mer).

Supplementary Table 1. Steady-State kinetics of hSIRT1 catalytic domain in the absence or presence of CTR peptide.[a]

		K_M (μM)	
Group	k_{cat} (s^{-1})	Ac-p53(W5)[b]	NAD^{+}[c]
hSIRT1cd	0.29 ± 0.01	163 ± 8	988 ± 73
hSIRT1cd + CTR	0.54 ± 0.01	37 ± 2.5	180 ± 14

[a] Data from PNC1/GDH assay.
[b] NAD^+ concentration fixed at 1 mM.
[c] Ac-p53(W) concentration fixed at 500 μM.

Supplementary Table 2. Steady-state kinetics of mini-hSIRT1 constructs.[a]

	hSIRT1	k_{cat} (s^{-1})	K_M (μM) Ac-p53(W5)[b]	K_M (μM) NAD^{+c}
Full length hSIRT1	hSIRT1(1-747)	0.37 ± 0.01	3.7 ± 0.8	70 ± 6
Mini-hSIRT1 ΔCTR	hSIRT1(183-516)	0.38 ± 0.01	42 ± 4.9	769 ± 97
Mini-hSIRT1	hSIRT1 (183-516-$(GGGS)_2$-641-665)	0.44 ± 0.02	16 ± 2.2	82 ± 6
	hSIRT1 (183-516-(GS)-641-665)	0.54 ± 0.01	14 ± 1.1	78 ± 8
	hSIRT1 (183-505-$(GGGS)_2$-641-665)	0.54 ± 0.02	13 ± 2	112 ± 10
Mini-hSIRT1 ΔSBD	hSIRT1 (229-516-$(GGGS)_2$-641-665)	0.49 ± 0.01	13 ± 3	222 ± 25
	hSIRT1 (229-505-$(GGGS)_2$-641-665)	0.43 ± 0.02	11 ± 3	218 ± 34
Mini-hSIRT1 (E230K)	hSIRT1 (183-505-$(GGGS)_2$-641-665) (E230K)	0.65 ± 0.03	14 ± 3	76 ± 14
Mini-hSIRT1 (R446E)	hSIRT1 (183-505-$(GGGS)_2$-641-665) (R446E)	0.21 ± 0.01	30 ± 3	375 ± 24
Mini-hSIRT1 (R446E,E230K)	hSIRT1 (183-505-$(GGGS)_2$-641-665) (R446E,E230K)	0.30 ± 0.01	33 ± 2	423 ± 42
Mini-hSIRT1 (R446F)	hSIRT1 (183-505-$(GGGS)_2$-641-665) (R446F)	0.19 ± 0.01	13 ± 1.3	123 ± 8
Mini-hSIRT1 (P231G,P232G)	hSIRT1 (183-505-$(GGGS)_2$-641-665) (P231G,P232G)	0.65 ± 0.01	23.4 ± 1	273 ± 26

[a] Data from PNC1/GDH assay.
[b] NAD^+ concentration fixed at 2 mM.
[c] Ac-p53(W) concentration fixed at 400 μM.

Supplementary Table 3. Inhibition of wild-type and mini-hSIRT1 constructs.

	IC$_{50}$ (μM)[a]		
hSIRT1	EX-527	TFA-p53 7-mer[b]	NAM
Full length	0.140 ± 0.020	0.680 ± 0.070	92 ± 6
hSIRT1 (183-516-(GS)-641-665)	0.116 ± 0.036	1.765 ± 0.158	62 ± 5
hSIRT1 (183-505-(GGGS)$_2$-641-665)	0.096 ± 0.008	1.318 ± 0.129	70 ± 5

[a] Data from OAcADPr assay using the Ac-p53(W5) substrate.
[b] TFA-p53 peptide sequence: Ac-RHKK(TFA)L-Nle-F-NH_2.

Supplementary Table 4. Steady-state substrate substrate kinetics for wild-type and mutant full-length hSIRT1.

		K_M (μM)	
hSIRT1	k_{cat} (s^{-1})[a]	Ac-p53(W5)[a]	NAD^{+}[a]
wild-type	0.37 ± 0.01	3.7 ± 0.8	70 ± 6
T219A	0.35 ± 0.01	2.7 ± 0.5	45 ± 4
Q222A	0.34 ± 0.01	7.1 ± 2.3	35 ± 6
I223A	0.33 ± 0.02	2.5 ± 0.3	96 ± 14
V224A	0.31 ± 0.01	6.2 ± 0.7	43 ± 4
N226A	0.34 ± 0.02	1.8 ± 0.7	85 ± 9
I227A	0.36 ± 0.01	5.3 ± 0.7	43 ± 6
E230K	0.36 ± 0.01	7.0 ± 0.7	70 ± 5
E230A	0.41 ± 0.01	6.2 ± 0.6	57 ± 2
E230Q	0.38 ± 0.01	3.2 ± 0.7	99 ± 17
I223R	0.27 ± 0.01	3.5 ± 0.5	91 ± 13

[a] Data from PNC1/GDH assay.

Supplementary Table 5. Inhibition of wild-type and mutant full-length hSIRT1.

	IC_{50} (μM)[a]		
hSIRT1	EX-527	TFA-p53 7-mer[b]	NAM
wild-type	0.140 ± 0.020	0.680 ± 0.070	92 ± 6
T219A	0.310 ± 0.040	0.490 ± 0.020	53 ± 4
Q222A	0.110 ± 0.010	0.630 ± 0.050	53 ± 5
I223A	0.180 ± 0.020	0.540 ± 0.150	108 ± 10
V224A	0.140 ± 0.010	0.860 ± 0.080	64 ± 3
N226A	0.190 ± 0.010	0.350 ± 0.040	100 ± 9
I227A	0.220 ± 0.020	0.940 ± 0.060	92 ± 4
E230K	0.150 ± 0.020	1.6 ± 0.3	54 ± 3
E230A	0.100 ± 0.030	1.3 ± 0.4	60 ± 3
E230Q	0.240 ± 0.020	0.860 ± 0.060	56 ± 3
I223R	0.210 ± 0.020	0.660 ± 0.060	87 ± 6

[a] Data from OAcADPr assay using the Ac-p53(W5) substrate.
[b] TFA-p53 peptide sequence: Ac-RHKK(TFA)L-Nle-F-NH_2.

Supplementary Table 6. Effect of hSIRT1 mutations on activator EC_{50} values.

compound:	1		3		4		5		6	
hSIRT1	EC_{50} (μM)	fold shift[a]	EC_{50} (μM)	fold shift[a]	EC_{50} (μM)	fold shift[a]	EC_{50} (μM)	fold shift[a]	EC_{50} (μM)	fold shift[a]
WT	0.30	1.00	0.77	1.00	0.44	1.00	1.04	1.00	1.53	1.00
T219A	1.00	3.30	2.38	3.10	1.95	4.38	1.54	1.48	N/A	N/A
Q222A	0.25	0.84	0.78	1.02	0.39	0.88	0.97	0.94	1.87	1.22
I223A	0.77	2.56	2.11	2.75	0.93	2.09	1.39	1.34	N/A	N/A
V224A	0.43	1.43	1.15	1.50	0.49	1.10	0.86	0.82	2.11	1.38
N226A	0.55	1.83	2.64	3.44	0.70	1.57	1.37	1.32	2.27	1.48
I227A	1.09	3.65	3.72	4.83	1.20	2.69	3.02	2.91	2.01	1.31
E230A	0.26	0.86	1.89	2.46	0.73	1.63	1.49	1.43	1.07	0.70
E230K	0.15	0.50	2.92	3.81	0.68	1.52	N/A	N/A	N/A	N/A
E230Q	0.37	1.23	2.15	2.80	0.75	1.68	N/A	N/A	N/A	N/A

compound:	7		8		9	
hSIRT1	EC_{50} (μM)	fold shift[a]	EC_{50} (μM)	fold shift[a]	EC_{50} (μM)	fold shift[a]
WT	2.20	1.00	0.48	1.00	0.77	1.00
T219A	8.83	4.01	3.96	8.26	4.12	5.36
Q222A	2.01	0.91	0.51	1.07	0.56	0.73
I223A	5.51	2.51	2.36	4.91	2.97	3.86
V224A	2.60	1.18	0.90	1.87	1.09	1.41
N226A	3.92	1.78	1.03	2.15	1.17	1.52
I227A	6.76	3.07	2.98	6.21	4.28	5.55
E230A	2.61	1.19	0.530	1.11	1.23	1.60
E230K	3.21	1.46	0.70	1.46	1.63	2.12
E230Q	3.36	1.53	0.58	1.21	1.34	1.74

[a] Fold shift = (mutant EC_{50}/wild-type EC_{50})

EC_{50} values determined from activation dose-response curves using **Eq. 1**. Activation was measured using the OAcADPr assay with Ac-p53(W5) substrate. N/A indicates that no reasonable curve fitting to generate EC50 values. No EC_{50} values for I223R were shown because no activation was observed.

Supplementary Table 7. Effect of hSIRT1 mutations on the maximum activation by STACs.

compound:	**1**		**3**		**4**		**5**		**6**	
hSIRT1	RV_{max}	fold shift[a]	RV_{max}	fold shift[a]	RV_{max}	fold shift[a]	RV_{max}	fold shift[a]	RV_{max}	fold shift[a]
WT	10.5	1.00	14.7	1.00	12.2	1.00	5.66	1.00	3.03	1.00
T219A	5.76	1.99	14.3	1.03	7.88	1.63	2.53	3.05	1.24	8.36
Q222A	7.58	1.44	10.9	1.39	8.12	1.57	3.98	1.57	2.85	1.10
I223A	4.52	2.69	9.85	1.55	12.25	1.00	5.68	1.00	1.42	4.87
V224A	9.56	1.11	12.9	1.15	10.37	1.20	4.96	1.18	3.09	0.97
N226A	7.82	1.39	11.2	1.34	6.94	1.89	3.69	1.73	3.23	0.91
I227A	5.22	2.25	6.14	2.67	5.63	2.42	2.29	3.62	3.04	0.99
E230A	2.64	5.81	6.15	2.66	3.72	4.12	1.60	7.79	1.31	6.60
E230K	1.39	24.3	3.83	4.84	1.93	12.1	1.20	23.0	1.09	21.9
E230Q	2.28	7.40	4.67	3.73	2.99	5.63	1.45	10.3	1.23	8.81

compound:	**7**		**8**		**9**	
hSIRT1	RV_{max}	fold shift[a]	RV_{max}	fold shift[a]	RV_{max}	fold shift[a]
WT	7.16	1.00	6.28	1.00	14.9	1.00
T219A	3.25	2.73	4.22	1.64	10.4	1.47
Q222A	7.27	0.98	5.32	1.22	10.4	1.48
I223A	1.89	6.90	6.45	0.97	8.00	1.98
V224A	9.58	0.72	7.71	0.79	14.5	1.03
N226A	5.68	1.32	4.95	1.34	13.4	1.12
I227A	8.25	0.85	5.98	1.06	12.5	1.20
E230A	2.72	3.58	3.21	2.39	6.91	2.35
E230K	1.71	8.67	1.99	5.36	2.96	7.06
E230Q	2.57	3.93	2.72	3.07	5.14	3.35

[a] Fold shift = (wild-type RV_{max}-1)/(mutant RV_{max}-1)

Maximum activation values (RV_{max}) determined from activation dose-response curves using **Eq. 1**. Activation was measured using the OAcADPr assay assay with Ac-p53(W5) substrate. No maximum activation values for I223R were shown because no activation was observed.

Supplementary Table 8. Steady-state kinetics for full-length hSIRT1 with FOXO-3a 21-mer [a].

hSIRT1	k_{cat} (s^{-1})	K_M peptide (μM)	K_M NAD^+ (μM)
wild type	0.39 ± 0.02	50 ± 6	280 ± 40
I223R	0.25 ± 0.01	90 ± 6	460 ± 50

[a] Data from PNC1/GDH assay.

SI Materials and Methods

Preparation of Compounds 1-9:

General Methods. Reagents were obtained from commercial sources and were used as received. All reactions were run under an inert atmosphere (N_2). Flash column chromatography was performed on silica gel, particle size 60 Å, mesh of 230−400 (Merck) under standard techniques. Unless otherwise indicated, chromatography refers to medium pressure chromatography performed on an ISCO Combiflash Rf or similar system. Purities of all compounds were determined by analytical HPLC using the area percentage method on the UV trace recorded at a wavelength of 254 nm, and compounds were found to have ≥ 95% purity unless otherwise specified. Analytical HPLC was performed on an Agilent 1100 Series HPLC equipped with a 3.5 μm Eclipse XDB-C18 (4.6 mm × 100 mm) column with the following conditions: CH_3CN/H_2O, modified with 0.1% formic acid mobile phase. Gradient elution: 5% CH_3CN hold (2 min), 5% to 95% CH_3CN gradient (11 min), 95% to 5% CH_3CN gradient (0.3 min), 5% CH_3CN hold (2.7 min), 15 min total run time with a flow rate of 0.8 ml/min. ^1H NMR spectra were obtained on a Bruker Advance III 300 MHz spectrometer and referenced to internal TMS (0.00 ppm), $CHCl_3$ (7.26 ppm), or DMSO (2.49 ppm). NMR spectral data are reported as follows: chemical shift (δ) in ppm, (multiplicity, coupling constants in Hertz, number of protons, assignment). Multiplicity abbreviations are as follows: s-singlet, d-doublet, t-triplet, q-quartet, m-multiplet, br-broad. Low Resolution Mass Spectral (LRMS) data were recorded using an Agilent 1100 series spectrometer integrated into an Agilent 1200 series HPLC system, in electrospray positive mode ionization. High Resolution Mass Spec (HRMS) was completed on a Waters qTOF Premiere Mass Spectrometer operating in W mode positive ionization with a resolving power of approximately 15,000. Flow injection was completed using a Waters nano-acquity LC. HRMS acceptable error is 3 mDa or 5 ppm, although most analyses are observed within 0.5 mDa with isotope fits in good agreement with the proposed structures.

Scheme S1, Preparation of Compound 1:

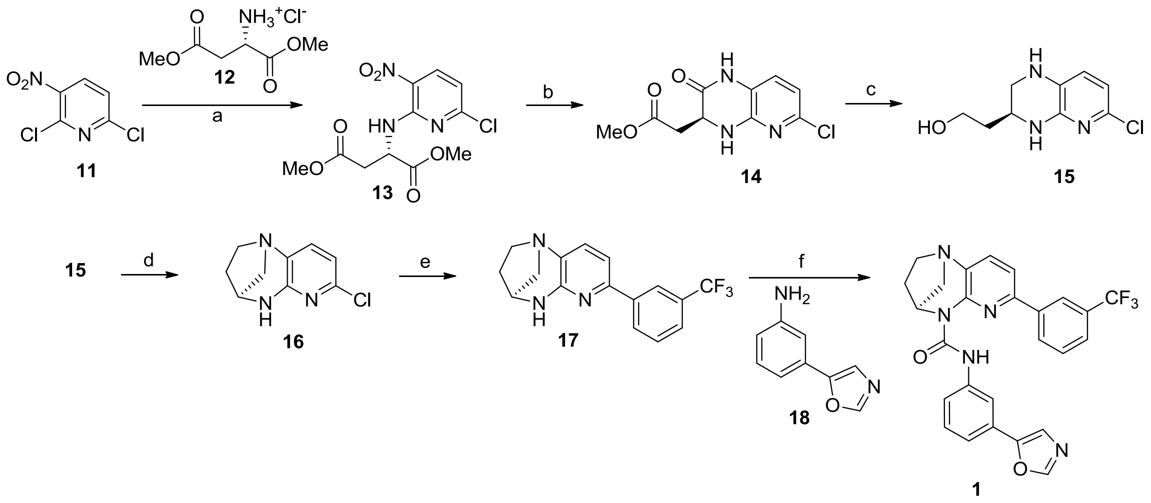

Reagents and conditions: a) $NaHCO_3$, THF, 40 °C; b) Fe, AcOH, IPA/Water 70°C, c) LAH, THF, 0-5 °C; d) $POCl_3$, CH_2Cl_2, TEA, e) 3-trifluorophenylboronic acid, $Pd(OAc)_2$, XPhos, Cs_2CO_3, Dioxane/Water; f) triphosgene, TEA, CH_2Cl_2, reflux.

***(S)*-Dimethyl 2-((6-chloro-3-nitropyridin-2-yl)amino)succinate (13).** To a 2 L flask equipped with a thermometer, a reflux condenser, and a mechanical stirrer was added 2,6-dichloro-3-nitropyridine (**11**; 100 g, 0.52 mol), *(S)*-aspartic acid dimethyl ester hydrochloride (**12**; 205 g, 1.04 mol), $NaHCO_3$ (174 g, 2.07 mol) and tetrahydrofuran (1 L). The reaction was stirred at 40 °C for 16 h, and was monitored for the disappearance of 2,6-dichloropyridine by HPLC. After the reaction was complete, the solids were filtered away and washed with ethyl acetate (3 × 300 mL). The combined filtrate and washings were concentrated to dryness, and the residue was taken up in 1 L of ethyl acetate. The solution was stirred with charcoal (200 g) at ambient temperature for 2 h, and the charcoal was filtered away and washed with additional ethyl acetate (3 x 200 mL). The combined filtrate and washings were concentrated in vacuo to obtain crude *(S)*-dimethyl 2-((6-chloro-3-nitropyridin-2-yl)amino)succinate (**13**; 180 g, >100%) as a yellow oil. This was used in the next step without further purification. LRMS (*m/z*): 318.0 $[M+H]^+$; HRMS (*m/z*): $[M+H]^+$ calcd for $C_{11}H_{13}N_3O_6Cl$, 318.0493; found, 318.0492; ^{1}H-NMR (300 MHz, d_6-DMSO): δ 9.00 (d, J = 7.9 Hz, 1H, -N*H*), 8.50 (d, J = 8.6 Hz, 1H), 6.92 (d, J = 8.6 Hz, 1H), 5.23 (m, J = 5.7, 7.9 Hz, 1H, -C*H*NH), 3.67 (s, 3H), 3.63 (s, 3H), 3.06 (m, J = 5.8 Hz, 2H, -CHC*H₂*); ^{13}C-NMR (APT) (75 MHz, d_6-DMSO): δ 170.93 (C), 170.65 (C), 154.65 (C), 150.59 (C), 138.82 (CH), 127.28 (C), 112.81 (CH), 52.23 (CH_3), 51.74 (CH_3), 50.20 (CH), 35.31 (CH_2).

***(S)*-Methyl 2-(6-chloro-2-oxo-1,2,3,4-tetrahydropyrido[2,3-*b*]pyrazin-3-yl)acetate (14).** To a 5L three necked flask equipped with a thermometer, a reflux condenser, and a mechanical stirrer was charged with crude *(S)*-dimethyl 2-((6-chloro-3-nitropyridin-2-yl)amino)succinate (**13**; 180 g, 0.52 mol), iron powder (146 g, 2.59 mol), 2-propanol (2 L) and water (700 mL). The mixture was stirred at 40 °C, then acetic acid (15.5 g, 0.259 mmol) was added at a rate sufficient to keep the internal temperature below 70 °C. The reaction was stirred at 70 °C for 30 min, HPLC indicated that the reaction was complete. The mixture was cooled to 40 °C, then Na_2CO_3 (165 g, 1.55 mol) was added, and the mixture was stirred for 1 h. The solids were filtered, and the solids were washed with tetrahydrofuran (3 x 500 mL). The combined filtrate and washings were concentrated in vacuo, then the residue was stirred in ethanol (1 L) for 12 hrs. The solid was filtered and washed with cold ethanol, and dried in vacuo to obtain *(S)*-methyl 2-(6-chloro-2-oxo-1,2,3,4-tetrahydropyrido[2,3-*b*]pyrazin-3-yl)acetate as an off-white solid (**14**; 91 g, 68%). LRMS (*m/z*): 256.0 $[M+H]^+$; HRMS (*m/z*): $[M+H]^+$ calcd for $C_{10}H_{11}N_3O_3Cl$, 256.0489; found, 256.0487; ^{1}H-NMR (300 MHz, d_6-DMSO): δ 10.55 (br s, 1H, -N*H*CO), 7.35 (br s, 1H, -N*H*CH), 6.92 (d, J = 7.9 Hz, 1H), 6.57 (d, J = 7.8 Hz, 1H), 4.43 (m, J = 1.4, 5.1 Hz, 1H, -NHC*H*), 3.57 (s, 3H, -CO_2*Me*), 2.79 (m, J = 5.1, 16.4 Hz, 2H, -CHC*H*₂); ^{13}C-NMR (APT) (75 MHz, d_6-DMSO): δ 170.32 (C), 164.96 (C), 146.13 (C), 140.32 (C), 122.41 (CH), 119.47 (C), 111.31 (CH), 51.81 (CH), 51.39 (CH_3), 37.01 (CH_2).

***(S)*-2-(6-Chloro-1,2,3,4-tetrahydropyrido[2,3-*b*]pyrazin-3-yl)ethanol (15).** A 5L 3-necked flask equipped with a mechanical stirrer, a reflux condenser, and a nitrogen inlet was charged with $LiAlH_4$ (60 g, 1.58 mol). The flask was cooled with an ice bath, and tetrahydrofuran (500 mL) was added. The stirred mixture was cooled to 0 °C, then a solution of *(S)*-methyl 2-(6-chloro-2-oxo-1,2,3,4-tetrahydropyrido[2,3-*b*]pyrazin-3-yl)acetate (**14**; 81 g, 0.32 mol) in

tetrahydrofuran (2 L) was added, while keeping the internal temperature below 5 °C. After the addition was complete, the reaction was heated at reflux for 16 h, while monitoring for the appearance of product by HPLC. The ester reduction occurred rapidly, while the lactam reduction required longer for complete reduction. The reaction was cooled to 5 °C, then water (60 mL) was added while keeping the internal temperature below 10 °C. After addition was complete, the reaction was stirred for 15 min. Then 15% (w/w) $NaOH_{(aq.)}$ (60 mL) was added while keeping the internal temperature below 5 °C. After addition was complete, the reaction was stirred for 15 min, then water (180 mL) was added and the mixture was stirred at ambient temperature for 1 h. The solids were filtered off and washed with tetrahydrofuran (3 x 150 mL). The filtrate and washings were concentrated in vacuo, then the solid residue was dried in vacuo to obtain *(S)*-2-(6-chloro-1,2,3,4-tetrahydropyrido[2,3-*b*]pyrazin-3-yl)ethanol as a brown solid (**15**; 55 g, 81%). LRMS (*m/z*): 214.1 $[M+H]^+$; HRMS (*m/z*): $[M+H]^+$ calcd for $C_9H_{13}N_3OCl$, 214.0747; found, 214.0743; ^{1}H-NMR (300 MHz, d_6-DMSO): δ 6.60 (br s, 1H, -N*H*CH(CH_2)$_2$OH), 6.58 (d, *J* = 7.8 Hz, 1H), 6.32 (d, *J* = 7.8 Hz, 1H), 5.69 (m, 1H, -N*H*CH_2), 4.57 (t, *J* = 5.0 Hz, 1H, -O*H*), 3.56 (m, *J* = 5.8 Hz, 2H, -C*H*$_2$OH), 3.47 (m, 1H, -NHC*H*(CH_2)$_2$OH), 3.22 (m, *J* = 2.7, 11.1 Hz, 1H, -NHC*H*H'), 2.84 (m, *J* = 1.6, 6.7, 11.1 Hz,1H, -NHCH*H'*), 1.65 (m, *J* = 6.7 Hz, 1H, -C*H*H'CH_2OH), 1.54 (m, *J* = 6.3 Hz, 1H, -CH*H'*CH_2OH); ^{13}C-NMR (APT) (75 MHz, d_6-DMSO): δ 146.75 (C), 134.44 (C), 128.20 (C), 118.97 (CH), 110.59 (CH), 57.97 (CH_2), 47.47 (CH), 43.99 (CH_2), 36.60 (CH_2).

(4*S*)-7-Chloro-2,3,4,5-tetrahydro-1,4-methanopyrido[2,3-*b*][1,4]diazepine (16). To a solution of *(S)*-2-(6-chloro-1,2,3,4-tetrahydropyrido[2,3-*b*]pyrazin-3-yl)ethanol (**15**; 50 g, 0.234 mol) in CH_2Cl_2 (500 mL) was added triethylamine (95 g, 0.936 mol). The mixture was stirred at ambient temperature until it was homogeneous, and then cooled to 0 °C. To the reaction mixture was added dropwise $POCl_3$ (54 g, 0.351 mol) while maintaining the temperature between 0 - 5 °C. Cooling was removed and the reaction was stirred at ambient temperature for 2 h, while monitoring for the disappearance of the starting alcohol by HPLC. After the reaction was complete, 1.2M $NaHCO_{3(aq.)}$ (200 mL) was added. The layers were separated and the aqueous layer was extracted with CH_2Cl_2. The combined CH_2Cl_2 layers were extracted with 1M $HCl_{(aq.)}$ (4 x 300 mL), and the combined HCl layers were adjusted to pH 8 with solid $NaHCO_3$. The resulting mixture was extracted with CH_2Cl_2 (4 x 300 mL), and this set of CH_2Cl_2 layers were dried (Na_2SO_4), filtered, and treated with charcoal (50 g). The mixture was stirred at ambient temperature for 3 h, filtered, and the charcoal was washed with CH_2Cl_2 (200 mL). The combined filtrate and wash solution were concentrated to dryness, and the solid residue was dried in vacuo to obtain (4*S*)-7-chloro-2,3,4,5-tetrahydro-1,4-methanopyrido[2,3-*b*][1,4]diazepine as an off-white crystalline solid (**16**; 30 g, 66%). LRMS (*m/z*): 196.1 $[M+H]^+$; HRMS (*m/z*): $[M+H]^+$ calcd for $C_9H_{11}N_3Cl$, 196.0642; found, 196.0637; ^{1}H-NMR (300 MHz, DMSO-d_6): δ 7.47 (br d, *J* = 4.5 Hz, 1H, -NH), 7.09 (d, *J* = 7.7 Hz, 1H), 6.39 (d, *J* = 7.7 Hz, 1H), 3.89 (m, *J* = 5.0 Hz, 1H, C*H*NH), 2.95-3.13 (m, 2H, -NC*H*$_2$$CH_2$CHNH), 2.77 (m, 2H, -NC*HH'*CHNH), 1.98 (m, *J* = 5.0 Hz, 1H, -NHCHC*HH'*CH_2N), 1.86 (m, *J* = 6.9 Hz, 1H, -NHCHC*HH'*CH_2N); ^{13}C-NMR (APT) (75 MHz, d_6-DMSO): δ 153.45 (C), 144.50 (C), 134.32 (CH), 133.19 (C), 109.73 (CH), 59.88 (CH_2), 53.07 (CH_2), 50.08 (CH), 38.38 (CH_2).

(4*S*)-7-(3-(Trifluoromethyl)phenyl)-2,3,4,5-tetrahydro-1,4-methanopyrido[2,3-*b*][1,4]diazepine (17). To a solution of (4*S*)-7-chloro-2,3,4,5-tetrahydro-1,4-methanopyrido[2,3-*b*][1,4]diazepine (**16**; 5.0 g, 25.6 mmol), 3-trifluoromethylphenylboronic acid (7.3 g, 38 mmol), $Pd(OAc)_2$ (0.14 g, 0.63 mmol), 2-dicyclohexylphosphino-2′,4′,6′-triisopropylbiphenyl (0.61 g,

1.3 mmol) and Cs_2CO_3 (24.9 g, 76.4 mmol) in a mixture of dioxane (100 mL) and water (10 mL) was heated at reflux for 2.5 hr and cooled to room temperature. The reaction mixture was filtered through celite and concentrated. The residue was diluted with ethyl acetate and the organic layer was washed with aqueous sat. $NaHCO_3$, water, brine, dried (Na_2SO_4) and concentrated to dryness. Purification by silica gel chromatography (50-100% ethyl acetate gradient in pentane) afforded (4*S*)-7-(3-(trifluoromethyl)phenyl)-2,3,4,5-tetrahydro-1,4-methanopyrido[2,3-*b*][1,4]diazepine as a white solid (**17**; 6.31g, 81%). LRMS (*m/z*): 305.1 $[M+H]^+$; HRMS (*m/z*): $[M+H]^+$ calcd for $C_{16}H_{15}N_3F_3$, 306.1218; found, 306.1219; ^{1}H-NMR (300 MHz, DMSO-d_6): δ 8.27 (s, 1H), 8.19 (d, J = 7.44 Hz, 1H), 7.69 (d, J = 7.9 Hz, 1H), 7.64 (t, J = 7.6 Hz, 1H), 7.27 (d, J = 4.5 Hz, 1H, -NH), 7.20 (d, J = 7.7 Hz, 1H), 7.09 (d, J = 7.7 Hz, 1H), 3.93 (m, J = 2.3 Hz, 1H), 3.00-3.17 (m, 2H), 2.85 (d, J = 11.2 Hz, 1H), 2.80 (dd, J = 2.0, 11.2 Hz, 1H), 1.96-2.07 (m, 1H), 1.85-1.96 (m, 1H); ^{13}C-NMR (APT) (75 MHz, DMSO-d_6): δ 153.31 (C), 149.41 (C), 140.10 (C), 134.64 (C), 132.42 (CH), 129.74 (CH), 129.54 (CH), 129.29 (q, J_{CF} = 31.5 Hz, C), 124.42 (q, J_{CF} = 3.7 Hz, CH), 124.33 (q, J_{CF} = 271.9 Hz, C), 122.41 (q, J_{CF} = 3.9 Hz, CH), 108.35 (CH), 59.85 (CH_2), 53.52 (CH_2), 50.31 (CH), 38.30 (CH_2).

(4*S*)-*N*-(3-(Oxazol-5-yl)phenyl)-7-(3-(trifluoromethyl)phenyl)-3,4-dihydro-1,4-methano-pyrido[2,3-*b*][1,4]diazepine-5(2*H*)-carboxamide (1). To a solution of (4*S*)-7-(3-(trifluoromethyl)phenyl)-2,3,4,5-tetrahydro-1,4-methanopyrido[2,3-*b*][1,4]diazepine (**17**; 3.05 g, 10 mmol) and triphosgene (2.37 g, 8.0 mmol) in CH_2Cl_2 (30 mL) was added triethylamine (4.17 mL, 30 mmol). The solution was heated to reflux for 1.5 hours, then 3-(oxazol-5-yl)aniline (**18**; 2.40 g, 15 mmol) was added as a solid. The reaction mixture heated at reflux for 1 hour, cooled to room temperature, diluted with CH_2Cl_2. The resulting organic layer was washed with sat. aqueous $NaHCO_3$, water, brine, dried (Na_2SO_4) and concentrated to dryness. During the aqueous workup the byproduct (1,3-bis(3-(oxazol-5-yl)phenyl)urea) formed a rag layer, which was removed by filtration. The crude product was purified by silica gel chromatography (0 to 6% MeOH gradient in CH_2Cl_2) to obtain **1** as a foam. The foam was sonicated in pentane, concentrated and dried under high vacuum to obtain (4*S*)-*N*-(3-(oxazol-5-yl)phenyl)-7-(3-(trifluoromethyl)phenyl)-3,4-dihydro-1,4-methanopyrido[2,3-*b*][1,4]diazepine-5(2*H*)-carboxamide as a free flowing white solid (**1**; 3.08 g, 63%). LRMS (*m/z*): 491.9 $[M+H]^+$; HRMS (*m/z*): $[M+H]^+$ calcd for $C_{26}H_{21}N_5O_2F_3$, 492.1647; found: 492.1646; ^{1}H-NMR (300 MHz, d_6-DMSO): δ 12.96 (s, 1H, NH), 8.46 (s, 1H), 8.26 (d, J = 7.7 Hz, 1H), 8.20 (m, 1H), 7.93 (m, 1H), 7.90 (d, J = 7.9 Hz, 1H), 7.82 (t, J = 7.7 Hz, 1H), 7.72 (d, J = 7.9 Hz, 1H), 7.65 (d, J = 7.9 Hz, 1H), 7.61 (s, 1H), 7.38-7.46 (m, 3H), 5.51 (dd, J = 2.9, 5.7 Hz, 1H), 3.05-3.25 (m, 3H), 2.98 (dd, J = 3.2, 12.0 Hz, 1H), 2.19-2.34 (m, 1H), 1.91-2.00 (m, 1H); ^{13}C-NMR (APT) (75 MHz, d_6-DMSO): δ 151.77 (C), 151.26 (CH assigned based on HSQC), 150.23 (C), 148.81 (C), 148.62 (C), 139.36 (C), 139.19 (C), 137.24 (C), 135.89 (CH), 130.65 (CH), 130.26 (CH), 129.91 (q, J_{CF} = 31.7 Hz, C), 129.59 (CH), 128.05 (C), 125.69 (m, J_{CF} = 4.2 Hz, CH), 123.97 (q, J_{CF} = 272.5 Hz, C), 122.91 (m, J_{CF} = 3.9 Hz, CH), 122.03 (CH), 119.28 (CH), 118.82 (CH), 115.59 (CH), 114.67 (CH), 58.73 (CH_2), 53.59 (CH_2), 51.66 (CH), 34.98 (CH_2).

<u>Preparation of Compound 2:</u>

The preparation procedure of Compound **2** has been reported previously[1]. LRMS (*m/z*): 384.0 $[M + H]^+$; HRMS (*m/z*): $[M + H]^+$ calcd for $C_{15}H_{22}N_5O_3S_2$, 384.1164; found: 384.1165. ^{1}H NMR (600MHz, d_6-DMSO): δ (ppm) 8.49 (s, 1H), 8.39 (br s, 1H), 8.03 (s, 1H), 7.86 (br s, 1H), 6.95 (br t, J=5.7 Hz, 1H), 4.70 (br d, J=13.2 Hz, 2H), 3.15-3.22 (m, 2H), 2.99 (q, J=6.7 Hz, 2H),

2.89 (s, 3H), 1.84 (br d, *J*=12.1 Hz, 2H), 1.71-1.81 (m, 1H), 1.42 (q, *J*=7.2 Hz, 2H), 1.14-1.22 (m, 2H); ^{13}C-NMR (APT) (151MHz, d_6-DMSO): δ (ppm) 162.37 (C), 160.70 (C), 157.03 (C), 154.53 (CH), 144.73 (C), 125.25 (CH), 115.73 (C), 46.11 (CH_2), 39.91 (CH_2), 39.11 (CH_3), 35.74 (CH_2), 32.65 (CH), 31.59 (CH_2).

Scheme S2, Preparation of Compound 3:

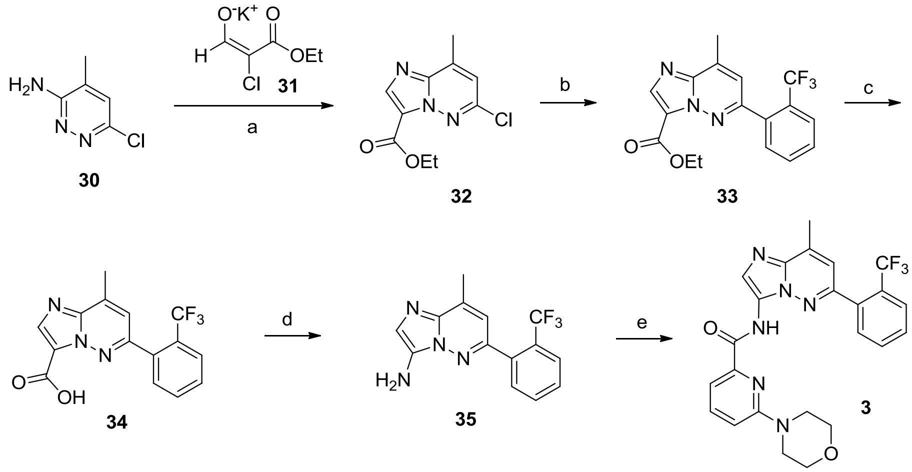

Reagents and conditions: a) conc. H_2SO_4, EtOH, reflux; b) 2-(trifluoromethyl)phenyl boronic acid, $Pd(PPh_3)_4$, Cs_2CO_3, Dioxane/EtOH/Water, reflux; c) NaOH, THF/CH_3OH/H_2O, 70 °C; d) DPPA, DMF, TEA, then H_2O, 90 °C e) 6-morpholinopicolinic acid, HATU, DIPEA, CH_3CN, reflux.

Ethyl 6-chloro-8-methylimidazo[1,2-*b*]pyridazine-3-carboxylatesuccinate (32). Conc. H_2SO_4 (1.0 mL, 18.00 mmol) was added to a suspension of the potassium salt of ethyl 2-chloro-3-oxopropanoate (**31**, 6.3 g, 33.40 mmol, *Tetrahedron*, **2000**, 56, 7915-7921) and an unresolved mixture of 6-chloro-4-methylpyridazin-3-amine and 6-chloro-5-methylpyridazin-3-amine (**30**; 2.38 g, 16.57 mmol, WO 2009/100375 A1) in EtOH (150 mL). The mixture was heated at reflux for 30 h then cooled to room temperature and concentrated to dryness. The crude residue was adsorbed onto silica gel and purified by column chromatography (15 to 85% ethyl acetate gradient in pentane) to give ethyl 6-chloro-8-methylimidazo[1,2-*b*]pyridazine-3-carboxylate as a white solid (**32**; 909 mg, 23%). LRMS (*m/z*) 240.1 $[M+H]^+$; HRMS (*m/z*): $[M+H]^+$ calcd for $C_{10}H_{11}N_3O_2Cl$, 240.0540; found: 240.0539; ^{1}H-NMR (300 MHz, $CDCl_3$): δ 8.30 (s, 1H), 7.10 (q, *J* = 1.1 Hz, 1H), 4.45 (q, *J* = 7.1 Hz, 2H), 2.70 (d, *J* = 1.1 Hz, 3H), 1.43 (t, *J* = 7.1 Hz, 3H). ^{13}C-NMR (APT) (75 MHz, $CDCl_3$): δ 158.61 (C), 148.50 (C), 142.09 (C), 140.17 (CH), 139.62 (C), 120.85 (C), 120.67 (CH), 61.21 (CH_2), 16.78 (CH_3), 14.55 (CH_3). Ethyl 6-chloro-7-methylimidazo[1,2-*b*]pyridazine-3-carboxylate was also isolated as a white solid (513 mg, 13 %). LRMS (*m/z*) 240.1 $[M+H]^+$; HRMS (*m/z*): $[M+H]^+$ calcd for $C_{10}H_{11}N_3O_2Cl$, 240.0540; found: 240.0540; ^{1}H-NMR (300 MHz, $CDCl_3$): δ 8.30 (s, 1H), 7.88 (q, *J* = 1.1 Hz, 1H), 4.44 (q, *J* = 7.1 Hz, 2H), 2.51 (d, *J* = 1.1 Hz, 3H), 1.42 (t, *J* = 7.1 Hz, 3H); ^{13}C-NMR (APT) (75 MHz, $CDCl_3$): δ 158.56 (C), 150.28 (C), 142.00 (C), 140.90 (CH), 130.97 (C), 125.89 (CH), 119.91 (C), 61.14 (CH_2), 20.18 (CH_3), 14.57 (CH_3).

Ethyl 8-methyl-6-(2-(trifluoromethyl)phenyl)imidazo[1,2-*b*]pyridazine-3-carboxylate (33). (2-(Trifluoromethyl)phenyl)boronic acid (594 mg, 3.13 mmol), $Pd(PPh_3)_4$ (240 mg, 0.21 mmol), and Cs_2CO_3 (1400 mg, 4.16 mmol) were sequentially added to a de-gassed solution of ethyl 6-chloro-8-methylimidazo[1,2-*b*]pyridazine-3-carboxylate (**32**; 500 mg, 2.08 mmol) in a mixture of dioxane/EtOH/H_2O (30 mL, 22:4:4). The mixture was heated at reflux for 2 h, then cooled to room temperature and partitioned between brine and EtOAc. The organic layer was separated, washed with brine, dried ($MgSO_4$) and concentrated. The crude residue was purified by column chromatography (15 to 85% ethyl acetate gradient in pentane) to give ethyl 8-methyl-6-(2-(trifluoromethyl)phenyl)imidazo[1,2-*b*]pyridazine-3-carboxylate as a white solid (**33**; 621mg, 85%). LRMS (*m/z*) 350.1 $[M+H]^+$; HRMS (*m/z*): $[M+H]^+$ calcd for $C_{17}H_{15}N_3O_2F_3$, 350.1116; found, 350.1116; ^{1}H-NMR (300 MHz, $CDCl_3$): δ 8.38 (s, 1H), 7.81 (d, *J* = 7.2 Hz, 1H), 7.69 - 7.59 (m, 3H), 7.19 (s, 1H), 4.42 (q, *J* = 7.1 Hz, 2H), 2.75 (d, *J* = 0.7 Hz, 3H), 1.39 (t, *J* = 7.1 Hz, 3H); ^{13}C-NMR (APT) (75 MHz, $CDCl_3$): δ 158.94 (C), 153.72 (C), 142.63 (C), 140.56 (CH), 137.10 (C), 135.58 (q, J_{CF} = 2.0 Hz, C), 132.11 (CH), 132.08 (CH), 129.77 (CH), 129.14 (q, J_{CF} = 31.3 Hz, C), 126.96 (q, J_{CF} = 5.1 Hz, CH), 124.04 (q, J_{CF} = 274.0 Hz, CF_3), 120.79 (C), 120.61 (q, J_{CF}= 1.9 Hz, CH), 60.93 (CH_2), 16.91 (CH_3), 14.57 (CH_3).

8-Methyl-6-(2-(trifluoromethyl)phenyl)imidazo[1,2-*b*]pyridazine-3-carboxylic acid (34). NaOH (120 mg, 3.00 mmol) was added to a solution of ethyl 8-methyl-6-(2-(trifluoromethyl)phenyl)imidazo[1,2-*b*]pyridazine-3-carboxylate (**33**; 200 mg, 0.57 mmol) in THF/CH_3OH/H_2O (15 mL, 1:1:1). The mixture was heated at 70 °C for 1.5 h then cooled to room temperature and concentrated. Water was added, followed by 3M $HCl_{(aq.)}$ until pH = 3. The resulting solid was collected by filtration rinsed with H_2O and dried under vacuum to give 8-methyl-6-(2-(trifluoromethyl)phenyl)imidazo[1,2-*b*]pyridazine-3-carboxylic acid as a white solid which was used without further purification (**34**; 166 mg, 90%). LRMS (*m/z*) 322.1 $[M+H]^+$; HRMS (*m/z*): $[M+H]^+$ calcd for $C_{15}H_{11}N_3O_2F_3$, 322.0803; found, 322.0802; ^{1}H-NMR (300 MHz, $CDCl_3$): δ 8.56 (s, 1H), 7.88 (dd, *J* = 6.8, 1.9 Hz, 1H), 7.77 - 7.68 (m, 2H), 7.56 (dd, *J* = 6.6, 1.7 Hz, 1H), 7.27 (s, 1H), 2.82 (d, *J* = 0.7 Hz, 3H); ^{13}C-NMR (APT) (75 MHz, $CDCl_3$): δ 158.41 (C), 153.33 (C), 141.51 (C), 141.45 (CH), 140.13 (C), 133.75 (q, J_{CF} = 2.0 Hz, C), 132.46 (CH), 131.53 (CH), 130.66 (CH), 129.10 (q, J_{CF} = 31.3 Hz, C), 127.36 (q, J_{CF} = 5.1 Hz, CH), 123.84 (q, J_{CF} = 274.9 Hz, CF_3), 120.69 (q, J_{CF} = 1.4 Hz, CH), 119.81 (C), 17.08 (CH_3).

8-Methyl-6-(2-(trifluoromethyl)phenyl)imidazo[1,2-*b*]pyridazin-3-amine (35). Diphenyl phosphorylazide (145 µl, 0.67 mmol) was added to a solution of 8-methyl-6-(2-(trifluoromethyl)phenyl)imidazo[1,2-*b*]pyridazine-3-carboxylic acid (**34**; 143 mg, 0.45 mmol) and TEA (93 µl, 0.67 mmol) in DMF (5 mL). The mixture was stirred at room temperature for 2 h, then H_2O (250 µl) was added and stirring was continued for 2 h at 90 °C. The mixture was poured into H_2O and extracted with CH_2Cl_2. The combined organics were washed with brine, dried ($MgSO_4$) and concentrated. The crude residue was purified by column chromatography (25 to 100% ethyl acetate gradient in pentane) to give 8-methyl-6-(2-(trifluoromethyl)phenyl)imidazo[1,2-*b*]pyridazin-3-amine (**35**; 79 mg, 60%). LRMS (*m/z*) 293.1 $[M+H]^+$; HRMS (*m/z*): $[M+H]^+$ calcd for $C_{14}H_{12}N_4F_3$, 293.1014; found, 293.1015; ^{1}H-NMR (300 MHz, $CDCl_3$): δ 7.83 (d, *J* = 7.3 Hz, 1H), 7.69 - 7.54 (m, 3H), 7.26 (s, 1H), 6.81 (s, 1H), 4.07 (br s, 2 H), 2.68 (s, 3H). ^{13}C-NMR (APT) (75 MHz, $CDCl_3$): δ 151.90 (C), 136.59 (C), 136.35 (q, J_{CF} = 1.8 Hz, C), 134.48 (C), 133.09 (C), 131.91 (CH), 131.65 (CH), 129.42 (CH), 129.06 (q, J_{CF} = 30.9 Hz, C), 126.92 (q, J_{CF} = 5.1 Hz, CH), 124.02 (q, J_{CF} = 271.4 Hz, CF_3), 117.14 (CH), 115.06 (q, J_{CF} = 1.6 Hz, CH), 16.38 (CH_3).

***N*-(8-Methyl-6-(2-(trifluoromethyl)phenyl)imidazo[1,2-*b*]pyridazin-3-yl)-6-morpholinopicolinamide (3).** A solution of 8-methyl-6-(2-(trifluoromethyl)phenyl)imidazo[1,2-*b*]pyridazin-3-amine (**35**; 197 mg, 0.67 mmol), 6-morpholinopicolinic acid (210 mg, 1.01 mmol), DIPEA (234 µl, 1.35 mmol) and HATU (513 mg, 1.35 mmol) in CH_3CN (15 mL) was heated at 75 °C for 16 h. The reaction mixture was partitioned between EtOAc and water. The organic layer was washed with brine, dried ($MgSO_4$) and concentrated. The crude residue was purified by column chromatography (50 to 100% ethyl acetate gradient in pentane) then recrystallized from CH_3CN to give *N*-(8-methyl-6-(2-(trifluoromethyl)phenyl)imidazo[1,2-*b*]pyridazin-3-yl)-6-morpholinopicolinamide (**2**; 254 mg, 78%). mp 180.2-181.6 °C. LRMS (*m/z*) 483.2 $[M+H]^+$; HRMS (*m/z*): $[M+H]^+$ calcd for $C_{24}H_{22}N_6O_2F_3$, 483.1756; found, 483.1756; ^{1}H-NMR (300 MHz, $CDCl_3$): δ 10.91 (s, 1H), 8.34 (s, 1H), 7.87 (dd , J = 7.2, 1.9 Hz, 1H), 7.73 - 7.63 (m, 4H), 7.56 (dd, J = 7.0, 1.7 Hz, 1H), 7.00 (s, 1H), 6.83 (dd, J = 8.2, 0.9 Hz, 1H), 3.65 - 3.62 (m, 4H), 3.49 - 3.46 (m, 4H), 2.79 (s, 3H); ^{13}C-NMR (APT) (75 MHz, $CDCl_3$): δ 161.39 (C), 158.12 (C), 152.21 (C), 146.81 (C), 139.32 (CH), 137.63 (C), 136.09 (q, J_{CF} = 2.2 Hz, C), 134.75 (C), 131.96 (CH), 131.55 (CH), 129.71 (CH), 129.13 (q, J_{CF} = 30.3 Hz, C), 126.86 (q, J_{CF} = 5.2 Hz, CH), 125.70 (C), 123.97 (q, J_{CF} = 271.3 Hz, CF_3), 121.70 (CH), 116.69 (q, J_{CF} = 1.1 Hz, CH), 112.51 (CH), 110.75 (CH), 66.67 (CH_2), 45.34 (CH_2), 16.63 (CH_3).

Scheme S3, Preparation of Compound 4:

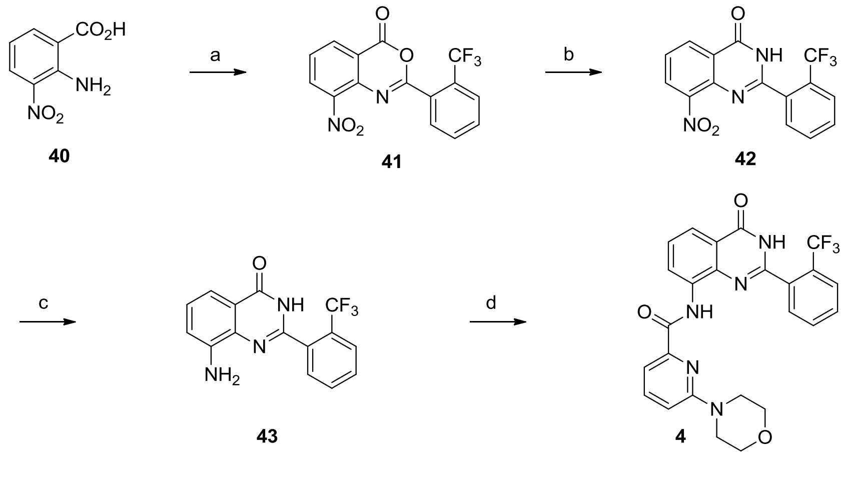

Reagents and conditions: a) RCOCl, CH_2Cl_2, TEA, 0°C then AC_2O, 50°C; b) NH_4OAc, AcOH, 120°C; c) Pd/C 10 wt%, cyclohexene, reflux; d) 6-morpholinopicolinic acid, HATU, DIPEA, DMF, 55°C.

8-nitro-2-(2-(trifluoromethyl)phenyl)-4H-benzo[d][1,3]oxazin-4-one (41): 2-trifluoromethylbenzoyl chloride (8.30 g, 40.00 mmol) was added to a solution of 2-amino-3-nitrobenzoic acid (**40**, 5.46 g, 30.00 mmol) and Et_3N (20.00 mL, 146.64 mmol) in CH_2Cl_2 (100 mL) at 0°C. The mixture was stirred for 16 h, slowly warming to room temperature. The reaction mixture was concentrated to half volume and Ac_2O (40 mL) was added. The reaction mixture was heated to 50°C for 3 h then cooled to room temperature and water (100 mL) was added. The mixture was extracted with CH_2Cl_2 and the combined organic layers were washed with sat. aq $NaHCO_3$, dried ($MgSO_4$) and concentrated to give 8-nitro-2-(2-(trifluoromethyl)phenyl)-4H-

benzo[d][1,3]oxazin-4-one (**41**, 10.10 g, 100 %) as a yellow solid which was used directly in next step with no further purification.

8-nitro-2-(2-(trifluoromethyl)phenyl)quinazolin-4(3H)-one (42): A mixture of 8-nitro-2-(2-(trifluoromethyl)phenyl)-4H-benzo[d][1,3]oxazin-4-one (**41**, 3.70 g, 11.00 mmol) and NH_4OAc (9.90 g, 129.00 mmol) in AcOH (80 mL) was heated at 120°C for 16 h. The reaction mixture was concentrated to dryness, diluted with CH_2Cl_2, washed with sat. aq $NaHCO_3$, dried ($MgSO_4$) and concentrated. The crude residue was purified by flash chromatography to give 8-nitro-2-(2-(trifluoromethyl)phenyl)quinazolin-4(3H)-one (**42**, 2.24 g, 61 %) as a yellow solid.

8-amino-2-(2-(trifluoromethyl)phenyl)quinazolin-4(3H)-one (43): A suspension of 8-nitro-2-(2-(trifluoromethyl)phenyl)quinazolin-4(3H)-one (**42**, 1.68 g, 5.00 mmol) and Pd/C (10 wt%, 1.55 g) in cyclohexene (30 mL) was heated at reflux for 8 hours. The catalyst was removed by filtration and the the mixture was concentrated to give 8-amino-2-(2-(trifluoromethyl)phenyl)quinazolin-4(3H)-one (**43**, 1.57 g, 100 %) as a yellow solid.

6-morpholino-N-(4-oxo-2-(2-(trifluoromethyl)phenyl)-3,4-dihydroquinazolin-8-yl)picolinamide (4): A mixture of 8-amino-2-(2-(trifluoromethyl)phenyl)quinazolin-4(3H)-one (**43**, 80 mg, 0.26 mmol), 6-morpholino-2-pyridinecarboxylic acid (65 mg, 0.53 mmol), HATU (190 mg, 0.50 mmol) and DIPEA (500 µL, 2.81 mmol) in anhydrous DMF (2.5 mL) was heated at 55°C for 16 h. The resulting mixture was poured into water (10 ml), extracted with CH_2Cl_2, dried ($MgSO_4$) and concentrated. The crude residue was purified by prep. TLC to give 6-morpholino-N-(4-oxo-2-(2-(trifluoromethyl)phenyl)-3,4-dihydroquinazolin-8-yl)picolinamide (**4,** 72 mg, 58%). LRMS (*m/z*): 496.2 $[M+H]^+$; calcd for $C_{25}H_{20}F_3N_5O_3$, 495.151824; ^{1}H NMR (600MHz, d_6-DMSO): δ (ppm) 12.90 (br s, 1H), 11.59 (s, 1H), 8.99 (dd, *J*=7.9, 1.1 Hz, 1H), 8.00 (br d, *J*=7.6 Hz, 1H), 7.87-7.94 (m, 1H), 7.86-7.91 (m, 1H), 7.84-7.93 (m, 1H), 7.83-7.88 (m, 1H), 7.78 (dd, *J*=8.5, 7.4 Hz, 1H), 7.62 (t, *J*=7.9 Hz, 1H), 7.49 (d, *J*=7.2 Hz, 1H), 7.09 (d, *J*=8.7 Hz, 1H), 3.23-3.28 (m, 4H), 3.18-3.23 (m, 4H); ^{13}C-NMR (APT) (151MHz, d_6-DMSO): δ (ppm) 162.11 (C), 160.98 (C), 157.55 (C), 152.23 (C), 146.81 (C), 139.31 (CH), 137.94 (C), 133.62 (C), 132.90 (C), 132.63 (CH), 130.73 (CH), 130.66 (CH), 127.39(CH), 126.93 (q, J_{CF} = 31.2 Hz, C), 126.42 (q, J_{CF} = 4.8 Hz, CH), 122.81 (q, J_{CF} = 273.9 Hz, C), 121.18 (C), 121.13 (CH), 119.55 (CH), 111.43 (CH), 111.07 (CH), 65.39 (CH_2), 44.47 (CH_2).

Scheme S4, Preparation of Compound 5:

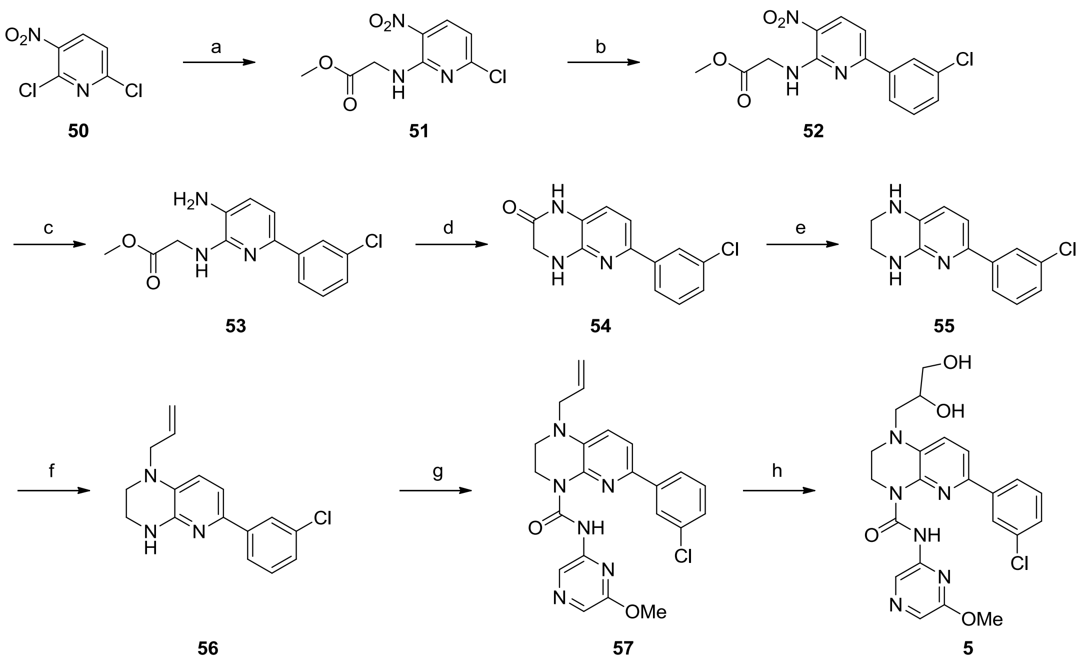

Reagents and conditions: (a) glycine methyl ester, DIPEA, DMF; (b) (3-chlorophenyl)boronic acid, $Pd(PPh_3)_4$, K_2CO_3, DME, 65 °C; (c) Pd/C, H_2, EtOH; (d) EtOH, 105 °C; (e) BH_3-Me_2S, THF; (f) 3-bromoprop-1-ene, Cs_2CO_3, DMF, 80 °C; (g) phenyl (6-methoxypyrazin-2-yl)carbamate, DMAP, CH_3CN, 80 °C; (h) NMO, OsO_4, acetone/H_2O.

Methyl (6-chloro-3-nitropyridin-2-yl)glycinate (51). Under a nitrogen atmosphere, a mixture of 2,6-dichloro-3-nitropyridine (**50**; 50 g, 259 mmol), glycine methyl ester (25 g, 285 mmol), and N,N-diisopropylethylamine (135 mL, 777 mmol) in DMF (2.6 L) was stirred at room temperature for 3 h. Saturated aqueous $NaHCO_3$ solution was added to the reaction mixture which was then extracted with ethyl acetate. The combined organic layers were washed with water and brine, then concentrated *in vacuo*. Purification by silica gel chromatography gave methyl (6-chloro-3-nitropyridin-2-yl)glycinate as yellow solid (**51**; 42 g, 66%).

Methyl (6-(3-chlorophenyl)-3-nitropyridin-2-yl)glycinate (52). A mixture of methyl (6-chloro-3-nitropyridin-2-yl)glycinate (**51**; 80 g, 308 mmol), (3-chlorophenyl)boronic acid (58 g, 370 mmol), $Pd(PPh_3)_4$ (17 g, 15 mmol) and K_2CO_3 (90 g, 616 mmol) in 1,2-dimethoxyethane (1.6 L) and water (12 mL) was stirred at 65 °C for 12 h. The solid was filtered and the filtrate was concentrated *in vacuo* to give a dark residue, which was triturated in petroleum ether/ethyl acetate (3:1) to afford methyl (6-(3-chlorophenyl)-3-nitropyridin-2-yl)glycinate as a yellow solid (**52**; 50 g, 48%).

6-(3-Chlorophenyl)-3,4-dihydropyrido[2,3-b]pyrazin-2(1H)-one (54). A mixture of methyl (6-(3-chlorophenyl)-3-nitropyridin-2-yl)glycinate (**52**; 50 g, 155 mmol) and palladium on carbon (5 g) in EtOH (1 L) was stirred under hydrogen for 16 h. The resulting mixture was filtered and

concentrated *in vacuo* to give methyl (3-amino-6-(3-chlorophenyl)pyridin-2-yl)glycinate (**53**) as a white solid, which was used in the next step without further purification. The solid was dissolved in EtOH (1.1 L), and stirred at 105 °C overnight. EtOH was removed under reduced pressure. The residue was dissolved in EtOH, activated charcoal was added and stirred at 60 °C for 3h, filtered, and crystallized to give 6-(3-chlorophenyl)-3,4-dihydropyrido[2,3-b]pyrazin-2(1H)-one as a white solid (**54**; 30 g, 77% over 2 steps).

6-(3-Chlorophenyl)-1,2,3,4-tetrahydropyrido[2,3-b]pyrazine (55). To a cooled solution of 6-(3-chlorophenyl)-3,4-dihydropyrido[2,3-b]pyrazin-2(1H)-one (**54**; 30 g, 116 mmol) in THF (500 mL) was added borane dimethyl sulfide complex solution (10 M in THF, 35 mL) at 0 °C. The reaction mixture was stirred at room temperature overnight. The reaction mixture was quenched with 1N HCl and stirred at 70 °C for 1 h. The pH of the reaction mixture was adjusted to 8 with saturated aqueous $NaHCO_3$ solution and extracted with ethyl acetate. The organic layer was dried over Na_2SO_4, concentrated *in vacuo* and purified by silica gel chromatography (eluent system MeOH : CH_2Cl_2 = 1 : 40) to give 6-(3-chlorophenyl)-1,2,3,4-tetrahydropyrido[2,3-b]pyrazine as a yellow solid (**55**; 14 g, 49%).

1-Allyl-6-(3-chlorophenyl)-1,2,3,4-tetrahydropyrido[2,3-b]pyrazine (56). A mixture of 6-(3-chlorophenyl)-1,2,3,4-tetrahydropyrido[2,3-b]pyrazine (**55**; 5.0 g, 20.3 mmol), allyl bromide (2.7 g, 24.4 mmol) and Cs_2CO_3 (6.6 g, 20.3 mmol) in DMF (50 mL) was stirred at 80 °C overnight. Saturated aqueous NH_4Cl (10 mL) and water (50 mL) were added to the reaction mixture, which was then extracted with ethyl acetate. The combined organic layers were washed with water, brine and dried, concentrated *in vacuo*. Purification by silica gel chromatography (eluent system petroleum ether : CH_2Cl_2 = 5 : 3) gave 1-allyl-6-(3-chlorophenyl)-1,2,3,4-tetrahydropyrido[2,3-b]pyrazine as a yellow solid (**56**; 2.0 g, 34%). LRMS (*m/z*) 286 $[M+H]^+$; ^{1}H-NMR (300 MHz, $CDCl_3$): δ 7.86 - 7.69 (m, 1H), 7.31 - 6.67 (m, 4H), 5.89 - 5.81(m, 1H), 5.28 - 5.09(m, 3H), 3.87 - 3.85(m, 2H), 3.55 - 3.53(m, 2H), 3.36 - 3.33(m, 2H).

1-Allyl-6-(3-chlorophenyl)-N-(6-methoxypyrazin-2-yl)-2,3-dihydropyrido[2,3-b]pyrazine-4(1H)-carboxamide (57). A mixture of 1-allyl-6-(3-chlorophenyl)-1,2,3,4-tetrahydropyrido[2,3-b]pyrazine (**56**; 73 mg, 0.25 mmol), phenyl (6-methoxypyrazin-2-yl)carbamate (109 mg, 0.51 mmol) and 4-(dimethylamino)pyridine (37 mg, 0.31 mmol) in acetonitrile (5 mL) was stirred at 80 °C overnight. The reaction mixture was directly loaded onto a preparatory thin-layer-chromatography plate and eluted with ethyl acetate/petroleum ether (3:1) to give 1-allyl-6-(3-chlorophenyl)-N-(6-methoxypyrazin-2-yl)-2,3-dihydropyrido[2,3-b]pyrazine-4(1H)-carboxamide as a white solid (**57**; 100 mg, 90%).

6-(3-Chlorophenyl)-1-(2,3-dihydroxypropyl)-N-(6-methoxypyrazin-2-yl)-2,3-dihydropyrido[2,3-b]pyrazine-4(1H)-carboxamide (5). To a solution of 1-allyl-6-(3-chlorophenyl)-N-(6-methoxypyrazin-2-yl)-2,3-dihydropyrido[2,3-b]pyrazine-4(1H)-carboxamide (**57**; 99 mg, 0.23 mmol) and 4-methylmorpholine N-oxide (106 mg, 0.91 mmol) in acetone/H_2O (8:1, 9 mL) was added an aqueous solution of osmium tetroxide (0.15 M, 100 μL). The mixture

was stirred at room temperature overnight. Saturated aqueous Na_2SO_3 solution was added and stirred for 2 h to quench the reaction. The resulting mixture was poured into water and extracted with ethyl acetate. The organic layer was dried over Na_2SO_4 and concentrated *in vacuo*. Recrystallization from MeOH/CH_2Cl_2 (10:1) – petroleum ether gave 6-(3-chlorophenyl)-1-(2,3-dihydroxypropyl)-N-(6-methoxypyrazin-2-yl)-2,3-dihydropyrido[2,3-b]pyrazine-4(1H)-carboxamide as a white solid (**5**; 26 mg, 24%). LRMS (*m/z*): 471.2 $[M+H]^+$; calcd for $C_{22}H_{23}ClN_6O_4$, 470.146931; ^{1}H NMR (600MHz, *d*$_6$-DMSO): δ (ppm) 12.76 (s, 1H), 8.91 (s, 1H), 7.99 (br d, *J*=7.9 Hz, 1H), 7.97 (s, 1H), 7.96 (br d, *J*=1.9 Hz, 1H), 7.70 (d, *J*=8.7 Hz, 1H), 7.47 (t, *J*=7.9 Hz, 1H), 7.38 (d, *J*=8.2 Hz, 1H), 7.30 (d, *J*=8.7 Hz, 1H), 4.94 (d, *J*=4.9 Hz, 1H), 4.77 (t, *J*=5.5 Hz, 1H), 4.14 (ddd, *J*=12.9, 5.9, 3.4 Hz, 1H), 3.96 (ddd, *J*=12.7, 7.0, 3.0 Hz, 1H), 3.79 (br d, *J*=2.6 Hz, 1H), 3.76 (s, 3H), 3.55-3.63 (m, 1H), 3.55-3.60 (m, 1H), 3.50-3.55 (m, 1H), 3.39-3.46 (m, 1H), 3.35-3.39 (m, 1H), 3.23-3.30 (m, 1H); ^{13}C-NMR (APT) (151MHz, *d*$_6$-DMSO): δ (ppm) 158.46 (C), 150.87 (C), 146.00 (C), 140.18 (C), 138.29 (C), 137.38 (C), 133.78 (C), 133.37 (C), 130.69 (CH), 127.96 (CH), 127.21 (CH), 126.08 (CH), 125.00 (CH), 123.64 (CH), 119.28 (CH), 117.43 (CH), 68.85 (CH), 63.71 (CH_2), 53.68 (CH_2), 53.14 (CH), 48.05 (CH_2), 38.91 (CH_2).

Preparation of Compound 6:

The preparation procedure of Compound **6** has been reported previously[2]. LRMS (*m/z*): 406.1 $[M+H]^+$; calcd for $C_{21}H_{19}N_5O_2S$, 405.125945; ^{1}H NMR (600MHz, *d*$_6$-DMSO): δ (ppm) 13.73 (br s, 2H), 8.03 (br d, *J*=7.6 Hz, 1H), 7.90 (br d, *J*=7.9 Hz, 1H), 7.87 (br s, 1H), 7.77 (br d, *J*=7.2 Hz, 1H), 7.57-7.68 (m, 1H), 7.52 (t, *J*=8.5 Hz, 1H), 7.48 (t, *J*=7.6 Hz, 1H), 7.28-7.41 (m, 1H), 7.20 (br d, *J*=7.9 Hz, 1H), 3.79-3.87 (m, 4H), 3.24-3.32 (m, 4H); ^{13}C-NMR (APT) (151MHz, *d*$_6$-DMSO): δ (ppm) 162.47 (C), 157.65 (C), 152.98 (C), 151.49 (C), 140.82 (C), 138.08 (CH), 135.28 (C), 129.99 (CH), 128.93 (C), 123.18 (CH), 122.98 (CH), 119.11 (C), 117.68 (CH), 117.64 (CH), 116.79 (CH), 114.12 (CH), 113.11 (CH), 66.00 (CH_2), 48.18 (CH_2).

Scheme S5, Preparation of Compound 7:

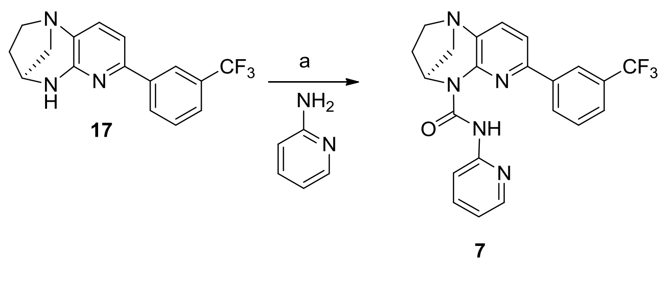

Reagents and conditions: a) triphosgene, TEA, THF, 60 °C.

(4S)-*N*-(Pyridin-2-yl)-7-(3-(trifluoromethyl)phenyl)-3,4-dihydro-1,4-methanopyrido[2,3-*b*] [1,4]diazepine-5(2*H*)-carboxamide (7). To a solution of (4*S*)-7-(3-(trifluoromethyl)phenyl)-

2,3,4,5-tetrahydro-1,4-methanopyrido[2,3-*b*][1,4]diazepine (**17**; 100 mg, 0.328 mmol) and Et_3N (160 µL, 1.15 mmol) in THF (4 mL) was added triphosgene (50 mg, 0.164 mmol). After stirring for 30 min at room temperature, 2-pyridylamine (92 mg, 0.983 mmol) was added. The reaction mixture was heated to 60 °C overnight. The reaction mixture was concentrated and the residue was taken up in CH_2Cl_2 (30 mL). The solution was washed with water and brine, dried ($MgSO_4$), filtered, and concentrated. Purification by silica gel chromatography afforded (4*S*)-*N*-(pyridin-2-yl)-7-(3-(trifluoromethyl)phenyl)-3,4-dihydro-1,4-methanopyrido[2,3-*b*][1,4]diazepine-5(2*H*)-carbox-amide (**7**; 86 mg, 62%). LRMS (*m/z*): 426.2 $[M+H]^+$; HRMS (*m/z*): $[M+H]^+$ calcd for $C_{22}H_{19}N_5OF_3$, 426.1542; found, 426.1544; ^{1}H-NMR (300 MHz, d_6-DMSO): δ 13.47 (s, 1H, -NH), 8.52 (m, 1H), 8.47 (m, *J* = 7.6 Hz, 1H), 8.30 (m, *J* = 1.1, 4.8 Hz, 1H), 8.13 (m, *J* = 0.9, 8.4 Hz, 1H), 7.80-7.87 (m, 3H), 7.77 (d, *J* = 8.1 Hz, 1H), 7.71 (d, *J* = 8.1 Hz, 1H), 7.10 (ddd, *J* = 1.0, 4.9, 7.3 Hz, 1H), 5.53 (dd, *J* = 3.0, 5.8 Hz, 1H), 3.17-3.28 (m, 1H), 3.05-3.16 (m, 2H), 2.96 (m, *J* = 3.3, 12.0 Hz, 1H), 2.18-2.32 (m, 1H), 1.89-2.02 (m, 1H); ^{13}C-NMR (APT) (75 MHz, d_6-DMSO): δ 152.16 (C), 150.84 (C), 148.55 (C), 148.36 (C), 148.09 (CH), 138.21 (CH), 137.36 (C), 136.06 (CH), 130.82 (CH), 129.93 (CH), 129.96 (q, J_{CF} = 31.7 Hz, C), 125.66 (q, J_{CF} = 3.7 Hz, CH), 124.10 (q, J_{CF} = 272.5 Hz, C), 123.23 (q, J_{CF} = 3.5 Hz, CH), 118.82 (CH), 115.45 (CH), 112.83 (CH), 58.68 (CH_2), 53.55 (CH_2), 51.53 (CH), 34.76 (CH_2).

<u>Scheme S6, Preparation of Compound 8:</u>

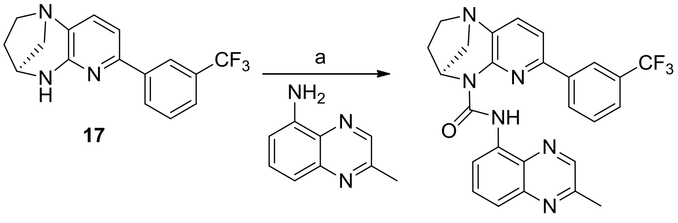

8

Reagents and conditions: a) triphosgene, TEA, CH_2Cl_2, reflux.

(4*S*)-*N*-(2-Methylquinoxalin-5-yl)-7-(3-(trifluoromethyl)phenyl)-3,4-dihydro-1,4-methano-pyrido[2,3-*b*][1,4]diazepine-5(2*H*)-carboxamide (8). To a solution of (4*S*)-7-(3-(trifluoromethyl)phenyl)-2,3,4,5-tetrahydro-1,4-methanopyrido[2,3-*b*][1,4]diazepine (**17**; 100 mg, 0.328 mmol) and Et_3N (136 µL, 1.0 mmol) in CH_2Cl_2 (15 mL) was added triphosgene (65 mg, 0.219 mmol). After stirring for 2.5 hr at room temperature, 2-methylquinoxalin-5-amine (52 mg, 0.327 mmol) was added. The reaction mixture was heated at reflux for 2 hours and cooled to room temperature. Saturated aqueous $NaHCO_3$ was added and the reaction mixture was extracted with CH_2Cl_2 (3x), and the combined organics were washed with brine, dried (Na_2SO_4) and concentrated. Purification by silica gel chromatography (20 to 100 % ethyl acetate in pentane gradient) afforded impure desired product (**8**; 80.6 mg, 83% purity). The impure material (**8**; 80 mg, 83% purity) was recrystallized from ethanol, dried under high vacuum to afford (4*S*)-*N*-(2-methylquinoxalin-5-yl)-7-(3-(trifluoromethyl)phenyl)-3,4-dihydro-1,4-methanopyrido[2,3-*b*][1,4]diazepine-5(2*H*)-carboxamide as a white solid (**8**; 31 mg, 19%, 99+% purity). LRMS (*m/z*): 491.2 $[M+H]^+$; HRMS (*m/z*): $[M+H]^+$ calcd for $C_{26}H_{22}N_6OF_3$, 491.1807; found, 491.1806; ^{1}H-NMR (300 MHz, d_6-DMSO): δ 13.58 (s, 1H, -NH), 8.57 (m, 1H), 8.28 (m, 1H), 8.06 (m, 1H), 7.75-7.90 (m, 2H), 7.68-7.77 (m, 2H), 7.60-7.66 (m, 2H), 7.49 (s, 1H), 5.57 (dd, *J* = 2.8, 5.5Hz,

1H), 3.19-3.30 (m, 1H), 3.07-3.19 (m, 2H), 2.99 (m, *J* = 3.0, 11.9 Hz, 1H), 2.54 (s, 3H), 2.22-2.35 (m, 1H), 1.93-2.06 (m, 1H); ^{13}C-NMR (APT) (75 MHz, d_6-DMSO): δ 154.10 (C), 151.22 (C), 149.80 (C), 148.71 (C), 143.36 (CH), 141.48 (C), 138.91 (C), 137.23 (C), 135.92 (C), 135.80 (CH), 131.33 (C), 131.21 (CH), 130.65 (CH), 129.75 (CH), 129.03 (q, J_{CF} = 31.7 Hz, C), 125.26 (q, J_{CF} = 3.7 Hz, CH), 124.37 (q, J_{CF} = 3.9 Hz, CH), 123.87 (q, J_{CF} = 270.1 Hz, C), 121.35 (CH), 116.37 (CH), 116.11 (CH), 58.78 (CH_2), 53.77 (CH_2), 52.10 (CH), 34.90 (CH_2), 21.75 (CH_3).

Scheme S7, Preparation of Compound 9:

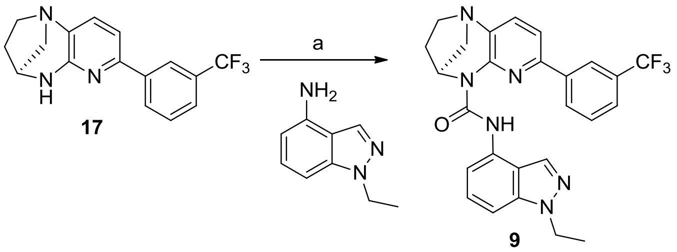

Reagents and conditions: a) triphosgene, TEA, CH_2Cl_2, reflux.

(4*S*)-*N*-(1-Ethyl-1*H*-indazol-4-yl)-7-(3-(trifluoromethyl)phenyl)-3,4-dihydro-1,4-methano-pyrido[2,3-*b*][1,4]diazepine-5(2*H*)-carboxamide (9). To a solution of (4*S*)-7-(3-(trifluoromethyl)phenyl)-2,3,4,5-tetrahydro-1,4-methanopyrido[2,3-*b*][1,4]diazepine (**17**; 100 mg, 0.328 mmol) in CH_2Cl_2 (15 mL) was added triphosgene (65 mg, 0.219 mmol) followed by Et_3N (136 µL, 1.0 mmol). After stirring for 1.5 hr at room temperature, 1-ethyl-1*H*-indazol-4-amine (45 mg, 0.279 mmol) was added. The reaction mixture was heated at reflux for 15 min, stirred at room temperature overnight, and returned to reflux for 2 hr. The reaction mixture was washed with saturated aq. $NaHCO_3$, dried (Na_2SO_4), filtered and concentrated to dryness. Purification by silica gel chromatography (0 to 100 % ethyl acetate in pentane gradient) afforded impure desired product (**9**; 62 mg, ~80% purity). The impure material (**9**; 62 mg, ~80% purity) was recrystallized from ethanol, dried under high vacuum to afford (4*S*)-N-(1-ethyl-1*H*-indazol-4-yl)-7-(3-(trifluoromethyl)phenyl)-3,4-dihydro-1,4-methano-pyrido[2,3-*b*][1,4]diazepine-5(2*H*)-carboxamide as a white solid (**9**, 24 mg, 17%, 99+% purity). LRMS (*m/z*): 493.2 $[M+H]^+$; HRMS (*m/z*): $[M+H]^+$ calcd for $C_{26}H_{24}N_6OF_3$, 493.1964; found, 493.1960; ^{1}H-NMR (300 MHz, d_6-DMSO): δ 12.94 (s, 1H, -NH), 8.18 (m, 1H), 8.09 (m, 1H), 7.83 (m, 1H), 7.73 (d, *J* = 8.0 Hz, 1H), 7.66 - 7.72 (m, 1H), 7.63 (d, *J* = 7.9 Hz, 1H), 7.56 (dd, *J* = 6.5 and 1.7 Hz, 1H), 7.40 (s, 1H), 7.29 - 7.39 (m, 2H), 5.54 (dd, *J* = 5.7, 2.9 Hz, 1H), 4.38 (q, *J* = 7.2 Hz, 2H), 3.06-3.30 (m, 3H), 2.99 (dd, *J* = 11.9 and 3.2 Hz, 1H), 2.20-2.34 (m, 1H), 1.93-2.07 (m, 1H), 1.34 (t, *J* = 7.2 Hz, 3H); ^{13}C-NMR (APT) (75 MHz, d_6-DMSO): δ 151.24 (C), 149.21 (C), 148.82 (C), 139.55 (C), 138.99 (C), 137.30 (C), 135.90 (CH), 130.97 (C), 130.81 (CH), 130.12 (CH), 129.89 (CH), 129.79 (q, J_{CF} = 30.6 Hz, C), 126.48 (CH), 125.69 (q, J_{CF} = 3.5 Hz, CH), 123.74 (q, J_{CF} = 271.4 Hz, C), 123.23 (q, J_{CF} = 3.9 Hz, CH), 116.66 (C), 115.86 (CH), 110.47 (CH), 104.68 (CH), 58.72 (CH_2), 53.56 (CH_2), 51.85 (CH), 43.00 (CH_2), 34.98 (CH_2), 14.68 (CH_3).

References and Notes:

1. Disch JS, Evindar G, Chiu CH, Blum CA, Dai H, Jin L, Schuman E, Lind KE, Belyanskaya SL, Deng J, *et al.* Discovery of Thieno[3,2-d]pyrimidine-6-carboxamides as potent inhibitors of SIRT1, SIRT2 and SIRT3. *Journal of medicinal chemistry* **56,** 3666-3679 (2013).
2. Dai H, Kustigian L, Carney D, Case A, Considine T, Hubbard BP, Perni RB, Riera TV, Szczepankiewicz B, Vlasuk GP, *et al.* SIRT1 activation by small molecules: kinetic and biophysical evidence for direct interaction of enzyme and activator. *J Biol Chem* **285,** 32695-32703 (2010).
